# A genetic common factor underlying self-reported math ability and highest math class taken

**DOI:** 10.1038/s41380-025-03237-0

**Published:** 2025-09-20

**Authors:** Alexandros Giannelis, Emily A. Willoughby, Tobias Edwards, Matt McGue, James J. Lee

**Affiliations:** 1 Department of Applied Economics, Erasmus School of Economics, Erasmus University Rotterdam, Rotterdam, The Netherlands; 2 Department of Psychology University of Minnesota Twin Cities, 75 East River Road, Minneapolis, MN 55455 USA

**Keywords:** Genetics, Psychology

## Abstract

While genetic influences on general intelligence have been well documented, less is known about the genetics underlying narrower abilities (“group factors”). By applying structural equation modeling to results from several genome-wide association studies (GWAS), most critically of self-reported math ability (*N* = 564 698) and highest math class taken (*N* = 430 445), we identified 53 single-nucleotide polymorphisms (SNPs) associated with a latent trait, orthogonal by design with general intelligence, approximating the group factor of quantitative ability. The genes near these SNPs implicated the biological process of neuron projection development, and the genome-wide pattern of gene-set enrichment affirmed the involvement of brain development and synaptic function. We calculated a number of genetic correlations with this quantitative factor, finding negative associations with both internalizing and externalizing disorders and positive associations with STEM occupations such as computer programming. These results provide further evidence for genetic influences on traits other than general factors in human behavioral variation, point to the mechanisms mediating these genetic influences on quantitative ability and interests, and affirm the relationships of the latter traits with a number of real-world outcomes.

Scores on different types of intelligence tests are always positively correlated, supporting their treatment as indicators of “general intelligence” (*g*) [1, 2]. A number of genome-wide association studies (GWAS) have focused on measures of overall intelligence, and because *g* is associated with the great bulk of the reliable variance in most such measures, these studies can be taken as providing molecular elucidation of mostly *g* regardless of how explicitly they have targeted this trait [3–6].

Broad factors other than *g* are often called “group factors” because each one is measured by a limited group or cluster of tests rather than all tests in general. Several taxonomies of group factors have been proposed [7–12]. An important criterion for judging the validity of a posited group factor is whether it provides incremental predictive value with respect to distinctive and important outcomes, and by this benchmark the two factors of verbal comprehension and spatial visualization—recognized in some form by all taxonomists—have proven their utility [13–19].

Quantitative ability is the capacity to draw logically necessary conclusions regarding number, change, and structure [20]. Some taxonomies classify quantitative ability as a facet of a more general “fluid reasoning” factor or crystallized knowledge. There are many reasons, however, to elevate quantitative ability to a group factor at the same level of prominence as verbal and spatial. A test of quantitative ability is often the best measure of *g* in its particular battery and furthermore might share little non-*g* variance with other reasoning tests [21, 22]. Regardless of whether a hierarchical or bifactor method is used in the factor analysis, this pattern embracing a test’s loadings on *g* and the quantitative factor renders the latter trait of considerable interest. It is also noteworthy that while other reasoning tests may not predict external criteria such as grades particularly well [23–25], tests of quantitative ability certainly do exhibit distinctive and important correlates, including success in STEM fields [16, 19, 26–28]. The quantitative and spatial factors should not be elided into a generic non-verbal ability, as each factor is associated with a distinct profile of interests, attitudes toward schooling, and vocational aspirations [29].

Twin studies suggest that at least some of the genetic influences on tests of quantitative reasoning or mathematical achievement are not shared with *g* [30–33]. In this study we attempted to identify specific sites in the human genome contributing to this unique variation. Such DNA-level analyses can uncover aspects of a trait that must remain obscure in traditional twin and family designs, such as clues to its underlying biological basis and the magnitudes of its associations with other traits that are rarely measured together with it in the same large sample. The observed-level phenotypes in our genome-wide association studies (GWAS) included self-reported math ability and highest math class taken [5]. Users of GWAS summary statistics are often limited to whatever can be practically measured in large-scale studies that are not dedicated to a specific research purpose, but nevertheless we believe that these two unconventional phenotypes should serve our purposes adequately as indicators of quantitative ability. We recognize that these two observed indicators may also reflect an interest in mathematics, distinct from an ability to do mathematics. Self-reported abilities have sometimes been reported to be more highly correlated with matching vocational interests than objectively measured abilities [34, 35], and interest may determine further education in mathematics as much as ability [36–38]. But related abilities, personality traits, and interests have often been considered together in previous work, as constellations of correlated and jointly developing attributes [39, 40], and our study might be regarded as a continuation of this approach.

To conduct the latent-level GWAS of the common factor underlying self-reported math ability and highest math class taken (Fig. 1), we turned to Genomic SEM, a software tool for applying factor and path analyses to genetic data [41], and applied a number of downstream analyses to the results.

**Fig. 1:**
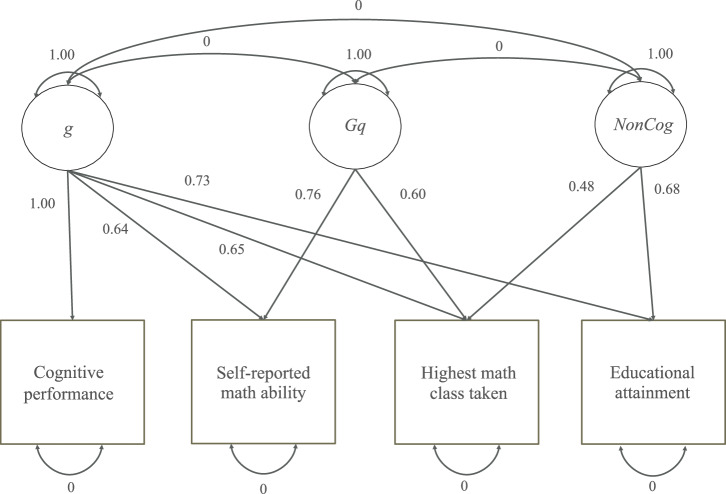
Path diagram depicting the standardized factor-analytic model used in this study. Each directed edge from factor to indicator is labeled with its associated factor loading. The residual variances of the indicators and covariances between the factors were fixed rather than estimated. Supplementary Table S1 provides the unstandardized solution. *g*, general intelligence; *Gq*, a symbol sometimes used in the literature for “quantitative knowledge” and adopted here to represent our attempt to capture the quantitative factor; *NonCog*, non-cognitive skills useful for attaining education.

## Materials and methods

A more complete description of Materials and Methods can be found in the Supplementary Information.

### Observed phenotypes

The GWAS of self-reported math ability (*N* = 564 698) and highest math class taken (*N* = 430 445) were conducted exclusively among research participants of 23andMe, Inc. who answered survey questions about their mathematical background. Our structural equation modeling also employed GWAS summary statistics of cognitive performance (CP) and educational attainment (EA) [5].

Self-reported math ability was assessed with the item: *How would you rate your mathematical ability?* Subjects responded to a 5-point scale ranging from *very poor* (1) to *excellent* (5). The mean of this item was 4.71, indicating a ceiling effect in the 23andMe sample. The standard deviation was 0.99. A study employing an extended twin design found that self-reported math ability is at least moderately heritable and correlated with objective ability measurements and grades in math (*r*_*g*_ > 0.7) [42]. Another study found a substantial phenotypic correlation (*r* > 0.6) between self-reported math ability and an objective achievement test [43]. Self-reported quantitative ability is considerably more correlated with measured quantitative ability than are other self-reported abilities with their corresponding measured abilities [35, 44]. These latter studies reported phenotypic correlations between self-reported numerical ability and scores on objective tests of number-series completion ranging from 0.41 to 0.53. The verbal and spatial correlations between self-report and objective scores ranged from 0.08 to 0.31, and the reported 95% confidence intervals did not overlap that of the numerical correlation.

### Confirmatory factor analysis and latent-level GWAS

We used the software tool Genomic SEM [41] to calculate the genetic covariance matrix of the phenotypes studied in the GWAS mentioned above. For this purpose Genomic SEM calls bivariate LDSC [45, 46]. A factor-analytic model was based on the genetic covariance matrix. Since the model in Fig. 1 contains four observed indicators and three common factors, it is not identified without further constraints. We chose as constraints the setting of all indicator residual variances to zero.

A GWAS of the quantitative factor in Fig. 1 was conducted with Genomic SEM. We used GCTA-COJO [47] to identify lead SNPs at a *P*-value threshold of 5 × 10^−8^. The COJO SNPs were then tested for a significant *Q*_SNP_ statistic. The threshold *P* < 5 × 10^−8^ was chosen as the criterion for classifying a SNP as being associated with the math indicators in a manner inconsistent with acting through the quantitative factor.

We searched the 53 lead SNPs in the NHGRI-EBI GWAS Catalog to check whether they have been associated with other traits by previous GWAS.

### Genetic correlations

We used bivariate LDSC to calculate genetic correlations between our quantitative factor and several behavioral, cognitive, psychiatric, and anthropometric traits. In order to establish the discriminant validity of the quantitative factor, we also calculated the genetic correlations with UKB job codes assumed to be related to general and group factors in the cognitive domain.

### Polygenic prediction

To convert our GWAS summary statistics of the quantitative factor into weights for polygenic scores (PGS), we first reran Genomic SEM with summary statistics of *g* [6] in the place of the summary statistics of CP [5]. We did this because our validation sample consisted of the Minnesota Twin Family Study and the Sibling Interaction and Behavior Study, both of which are being conducted by the Minnesota Center for Twin and Family Research (MCTFR) [48], and the MCTFR was a contributor to the meta-analysis of CP. To the resulting new summary statistics of the quantitative factor, we applied the software tool PRS-CS to generate the PGS weights [49].

Each complete unit in the validation sample was made up of two siblings (usually twins) and their parents. The total sample consisted of 9 067 individuals, belonging to 2 497 family units. Details about genotyping and quality control were provided in an earlier paper [50].

Our outcome measure was the third edition of the Wide Range Achievement Test (WRAT). The test has three components: Reading, Spelling, and Arithmetic. The offspring were tested on the WRAT during adolescence, between ages 13 and 21. There were 2 641 genotyped individuals in the offspring generation of European ancestry with available WRAT scores.

We repeated all of our PGS predictions except restricting observations to individuals with genotyped parents and adding the parental PGS as covariates. For a fixed value of the parental PGS, the PGS of the offspring vary randomly as a result of Mendelian segregation and thus provide a strong degree of causal inference [51–54].

### Biological annotation

We used stratified LD Score regression (S-LDSC) [55] to identify the tissues mediating the genetic effects of the SNPs affecting the quantitative factor. We used the precomputed stratified LD Scores for the Genotype-Tissue Expression (GTEx) [56] data supplied by the developers [57].

To prioritize likely causal genes mediating SNP effects, we turned to Polygenic Priority Score (PoPS) [58]. After considering the gene features used in the PoPS paper, we decided to use the same expression data from mice, the human GTEx data, and the reconstituted gene sets employed by the bioinformatic tool DEPICT [59]. Once in hand, the PoPS marginal and partial regression coefficients can be used as a form of gene-set enrichment analysis.

To confirm the robustness of our inferences based on the DEPICT reconstituted gene sets with quantitative membership scores, we turned to two methods relying on the current discrete versions of these gene sets. The first was the PANTHER overrepresentation test, which has been implemented as a web-based tool (https://www.geneontology.org). Our second method was the application of S-LDSC to calculate heritability enrichment.

## Results

### Genomic factor analysis

Table 1 presents the heritabilities of our indicators and their genetic correlations, as estimated by LD Score regression (LDSC) [45, 60]. The genetic correlations were all quite high, which suggests that much of the noise in an indicator contributed to the environmental term without substantially affecting the heritable component. The two indicators of the quantitative factor were highly correlated with cognitive performance (CP) (*r*_*g*_ > 0.6), highlighting the need to partial out CP from the indicators in order to carry out a GWAS of the residual quantitative factor. Highest math class taken was strongly correlated with educational attainment (EA) (*r*_*g*_ = 0.78), as expected; few high-school dropouts will successfully complete, say, complex analysis. This dependence points to the need to remove the variance shared with EA from highest math class taken to isolate the quantitative factor. The largest genetic correlation was between self-reported math ability and highest math class taken (*r*_*g*_ = 0.84), suggesting that these two variables share a source of variance measured by neither CP nor EA.

**Table 1:** LDSC heritabilities and genetic correlations of observed indicators.

	(1)	(2)	(3)	(4)
(1) Cognitive performance	0.19 (0.01)			
(2) Educational attainment	0.69 (0.02)	0.11 (0.02)		
(3) Self-reported math ability	0.60 (0.02)	0.49 (0.02)	0.16 (0.01)	
(4) Highest math class taken	0.64 (0.02)	0.78 (0.02)	0.84 (0.02)	0.17 (0.01)

Common-SNP heritabilities are on the diagonal, and other entries are genetic correlations. Block-jackknife standard errors are in parentheses.

With these considerations in mind, we adopted the factor-analytic model shown in Fig. 1. All four indicators were taken as indicators or effects of general intelligence, *g*. Highest math class taken and EA were taken as indicators of non-cognitive skills (*NonCog*), here defined to be a composite of all attributes important for success in education that is orthogonal to CP [61]. All estimated loadings handily exceeded the conventional salience threshold of 0.3. The two math indicators loaded nearly as strongly on their group factor as on *g* or even more so, a pattern usually not observed in bifactor-modeling studies with typical samples and measures [22, 62]. Our pattern of factor loadings may be owed to several features of our study. First, the genetic covariance between self-reported math ability and highest math class taken may have contributions from multiple sources, including not only abilities but interests as well. Second, because we had few indicators of any given factor, we fixed each indicator’s residual genetic variance to zero in order to achieve model identification. As a consequence, CP loaded on no other factor besides *g* in the model, effectively fixing its factor loading to unity, and this loading may not have been the only one in the model that was somewhat distorted.

Our factor model closely approximated the observed genetic covariance matrix, as evidenced by the fit indices: *χ*^2^(2) = 111.82, CFI = 0.996, SRMR = 0.0195.

### Multivariate GWAS of the quantitative factor

**Fig. 2:**
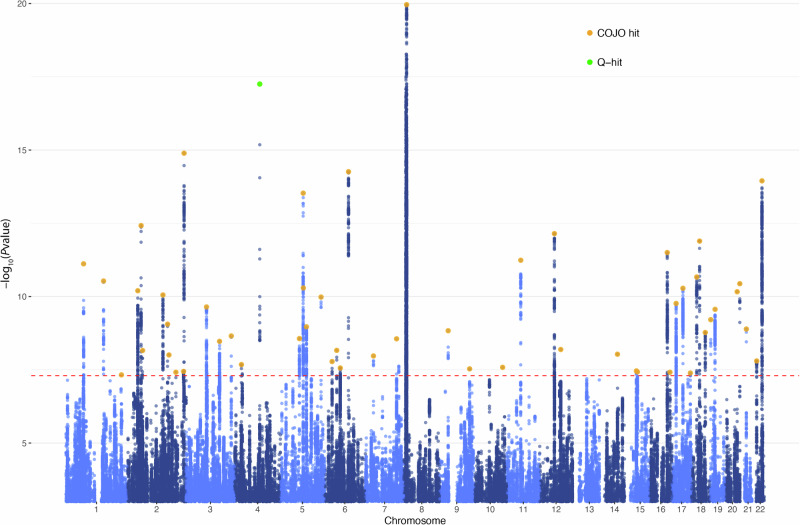
Genome-wide association study with the latent quantitative factor. Each data point represents −log_10_(*P* value) for the regression of the quantitative factor on the SNP whose location is given by the abscissa. Orange circles represent the 53 COJO hits. The one SNP that showed significant *Q*_SNP_ heterogeneity is colored in green. The red dashed line marks the threshold for genome-wide significance (*P* = 5 × 10^−8^).

By including SNP effects in our structural equation model (Supplementary Fig. S1), we were able to conduct a GWAS of the latent quantitative factor. Figure 2 displays the Manhattan plot. The mean *χ*^2^ over HapMap3 SNPs was 1.64, comparable to that obtained in previous GWAS yielding strong signals [63–65]. The LDSC intercept was 0.99 (s.e. = 0.01) and the attenuation ratio −0.01 (s.e. = 0.01), suggesting negligible bias from population stratification. Our analysis identified 53 SNPs conditionally and jointly associated with the quantitative factor (Supplementary Table S2).

We additionally used Genomic SEM to perform SNP-level tests of heterogeneity. For each lead SNP, we estimated a *Q*_SNP_ statistic to investigate whether the SNP’s associations with the indicators cannot be explained by the mediation of the quantitative factor. Only one lead SNP (rs13107325, a missense variant in *SLC39A8*) showed significant *Q*_SNP_ heterogeneity (*P* < 3 × 10^−15^). This SNP appears to be highly pleiotropic and has been implicated in multiple GWAS of physical and behavioral traits. Its associations with the two indicators of the quantitative factor were estimated to have opposite signs (highest math class taken, *Z* = − 3.56, *P* < 4 × 10^−4^; self-reported math ability, *Z* = 6.44, *P* < 2 × 10^−10^).

We performed a phenome-wide scan of the 53 lead SNPs—plus any SNPs in LD (*r*^2^ > 0.6) with them—in the NHGRI-EBI GWAS Catalog to look for associations with other traits (Supplementary Table S3). Of the 53 SNPs, 23 were significantly associated with the main indicator of the quantitative factor, self-reported math ability. From the same set of SNPs, 13 were associated with highest math class taken, 9 with cognitive performance, and 11 with educational attainment. These variants also showed a recurring pattern of associations with other phenotypes. The associations were mainly with traits of the internalizing spectrum (neuroticism, worry, anxiety, major depressive disorder); sleep (insomnia, chronotype, sleep duration); brain-related phenotypes (e.g., cortical surface area); and substance use (e.g., smoking initiation, alcohol use). Our analysis identified 16 novel SNPs, in the sense of not having been previously associated with any of the four indicators or any other cognitive traits. Of the 16 novel SNPs, four were significantly associated with other behavioral traits: rs10863150 with major depressive disorder, rs4459682 with insomnia, rs2572379 with neuroticism and smoking cessation, and rs10515368 with smoking initiation. Another novel SNP, rs57394143, was associated with thalamus volume.

### Genetic correlations

**Fig. 3:**
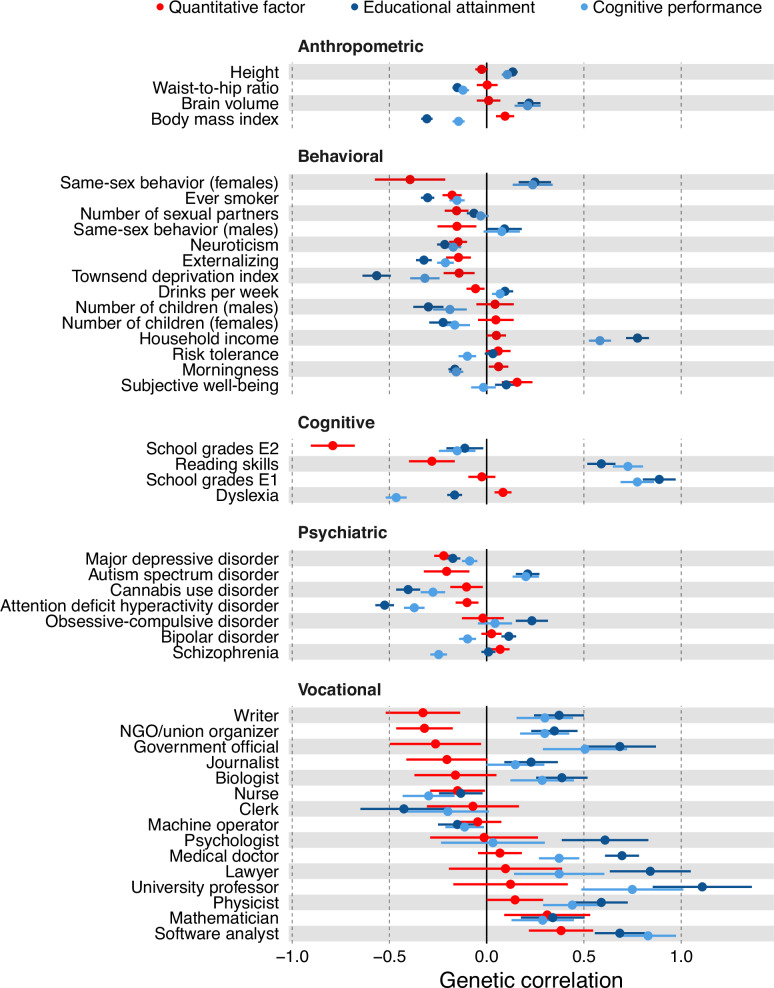
Genetic correlations of selected phenotypes with the quantitative factor, cognitive performance (CP), and educational attainment (EA). Error bars represent ±1.96-s.e. intervals. The results in numerical form are reported in Supplementary Table S4.

Figure 3 shows genetic correlations between the quantitative factor and a wide range of phenotypes. For comparison, we also present genetic correlations between those phenotypes and the two indicators (CP, EA) of other common factors in our model (Fig. 1).

The quantitative factor is uncorrelated with the first principal component of school grades (*r*_*g*_ = −0.02; s.e. = 0.03), which captures a general scholastic ability [66]. This result is reassuring, given that the factor is defined as orthogonal to general cognitive ability and non-cognitive educational skills. It is also correlated negatively with the second principal component of school grades (*r*_*g*_ = −0.80; s.e. = 0.05), an axis that reflects differences between language and math performance (with higher values indicating better language performance and lower values indicating better mathematical performance). There was a small but significantly positive correlation with dyslexia (*r*_*g*_ = 0.08; s.e. = 0.02).

A similar pattern was found when examining associations with job codes from the UK Biobank (UKB). The quantitative factor is correlated with employment as a mathematician (*r*_*g*_ = 0.31; s.e. = 0.11) and software engineer or programmer (*r*_*g*_ = 0.38; s.e. = 0.03). We did not observe any significant associations with being a university professor, medical doctor, life scientist, or psychologist, providing evidence for the discriminant validity of the quantitative factor. We did observe significantly negative correlations with intuitively verbal vocational categories, such as writer/poet (*r*_*g*_ = −0.32; s.e. = 0.09).

There were no significant associations between the quantitative factor and any anthropometric traits, except a small correlation with body mass index (*r*_*g*_ = 0.09; s.e. = 0.02). The quantitative factor is correlated negatively with attention deficit hyperactivity disorder (*r*_*g*_ = −0.10; s.e. = 0.02) and with the general factor of externalizing (*r*_*g*_ = −0.14; s.e. = 0.03). It is also correlated negatively with traits defining internalizing behavior, such as the general factor of neuroticism (*r*_*g*_ = −0.14; s.e. = 0.02) and major depressive disorder (*r*_*g*_ = −0.22; s.e. = 0.02). Like CP and EA, the quantitative factor was correlated negatively with neighborhood deprivation (*r*_*g*_ = −0.14; s.e. = 0.04). However, unlike CP and EA, it was also correlated negatively with autism spectrum disorder (*r*_*g*_ = −0.20; s.e. = 0.05). Although perhaps surprising, this finding was in accord with a recent meta-analysis reporting a negative association between autism spectrum disorder and quantitative reasoning that is not moderated by full-scale IQ [67]. Finally, there was a small but significantly positive association with schizophrenia (*r*_*g*_ = 0.07; s.e. = 0.02).

### Polygenic prediction

Polygenic scores (PGS) for the quantitative factor were used to predict student achievement in a holdout sample [48]. The PGS was a positive predictor of arithmetic achievement (*β* = 0.063; s.e. = 0.025; Δ*R*^2^ = 0.39%). In contrast, the PGS happened to have near-zero coefficients in the prediction of reading (*β* = −0.005; s.e. = 0.025; Δ*R*^2^ ≈ 0) and spelling achievement (*β* = 0.003; s.e. = 0.03; Δ*R*^2^ ≈ 0). This pattern of results persisted in the within-family analysis, consistent with an absence of strong confounding bias in the PGS weights. The full results are presented in Supplementary Table S5.

One study found several tests of quantitative ability to range in their loadings on their group factor from 0.29 to 0.46 [22]. It is reasonable to consider the lower end of this range because the elementary problems making up most of WRAT Arithmetic may have a different factorial composition than more advanced tests of quantitative reasoning. If we suppose that 9 percent of the variance in WRAT Arithmetic is attributable to our quantitative factor, that half of this variance is heritable, that half of the heritable variance is attributable to common SNPs, and that our GWAS was sufficiently powered to produce a PGS accounting for half of its potential variance in the limit of infinite sample size, then the PGS should account for something like 1 percent of the variance. If we assume that our estimate of the PGS coefficient is perhaps a standard error too low, then our predictive power does not seem too far off from what we can reasonably expect.

### Biological annotation

In many of our analyses, we employed stratified LD Score regression (S-LDSC) to estimate heritability enrichment. It is recommended that S-LDSC be used with a standard collection of control variables. The estimates associated with these variables can be interesting in their own right, and we give them in Supplementary Table S6. The most statistically significant enrichments were shown by annotations referring to evolutionary conservation, a pattern typical of traits that have been studied in GWAS [55, 65]. What the pattern means is that mutations affecting the quantitative factor (and other traits) tend to arise in functional regions of the genome, as evidenced by selection to maintain sequence similarity in distinct lineages, and once arisen may be deleterious.

**Fig. 4:**
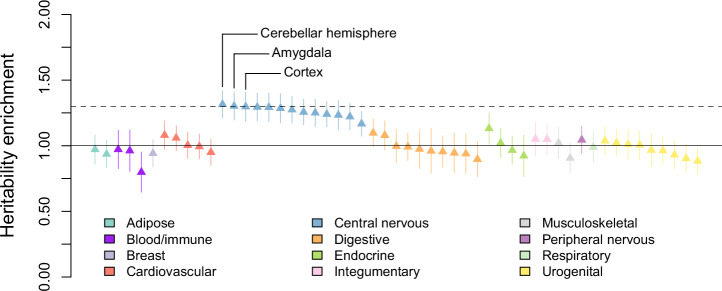
Heritability enrichment of Genotype-Tissue Expression (GTEx) tissues and cell types, as estimated by stratified LD Score regression (S-LDSC) applied to the GWAS summary statistics of the quantitative factor. The error bars are ±1.96-s.e. intervals. The height of the dashed horizontal line corresponds to 1.3-fold enrichment, which we consider to be a “large” effect size. Complete numerical results are given Supplementary Table S7.

Figure 4 shows that the top 13 GTEx tissues by heritability enrichment were, without exception, those of the central nervous system. None of the others showed a statistically significant positive enrichment. The two tissues clearing our benchmark effect size of 1.3-fold enrichment were *cerebellar hemisphere* (*P* < 10^−6^) and *amygdala* (*P* < 5 × 10^−8^). The estimated effect sizes of the top tissues were rather close, and not much should be read into their precise ranking. It is perhaps well to regard *amygdala* skeptically because multiple regression of MAGMA gene-prioritization score [68] on numerous predictors, including expression in other brain regions, left this annotation with a negative weight (Supplementary Table S8). In this latter analysis, *frontal cortex*, *anterior cingulate cortex*, *cortex*, *cerebellar hemisphere*, *cerebellum*, and *hippocampus* all retained positive weights. Overall, there can be no doubt that genetic variation affects the quantitative factor disproportionately through expression in regions of the brain subserving cognition.

To gain further insight into the neural underpinnings of the quantitative factor, we turned to the genes prioritized by the software tool PoPS (Supplementary Table S9] [58]. These genes were significantly overrepresented in the Gene Ontology biological process *regulation of neuron projection development* (8.4-fold enrichment, *P* < 10^−6^; Supplementary Table S10), defined as “any process that modulates the rate, frequency, or extent of … the progression of [axons or dendrites] over time” (https://amigo.geneontology.org/amigo/term/GO:0010975). The members of this set prioritized by PoPS are indicated in Supplementary Table S2. *SEMA6D* encodes a member of a well-known ligand family that binds to receptors in the growth cones of extending axons, typically repelling the axons toward regions of lower concentration. *EFNA5* encodes a member of another well-known ligand family implicated in axon guidance. Curiously, *SEMA6D* and the gene encoding the receptor of EFNA5 were also among the first genes prioritized for the phenotype of EA [64].

Because the quantitative factor shows no genetic correlation with brain volume (Fig. 3), it is reasonable to suspect that earlier stages of brain development (progenitor proliferation, neurogenesis) play a lesser role in the quantitative factor than they do in CP and EA. This inference fits well with the above findings regarding the development of axons and dendrites.

The above enrichment analysis implicating the growth of axons and dendrites during brain development drew only upon genes near the 53 significant lead SNPs. The most informative features in the PoPS procedure for prioritizing genes can also provide insight and happen to be identified by data from most of the protein-coding genes in the human genome without regard to a threshold of statistical significance in the GWAS. The features with the largest positive weights in the PoPS gene-prioritiziation run tended to be loadings on principal or independent components of gene expression in the mouse brain (Supplementary Table S8). We regard gene sets from databases such as Gene Ontology to be more biologically informative than expression data, and the top features of this type include *dendrite* (marginal *P* < 10^−15^), *presynaptic membrane* (marginal *P* < 10^−14^), and *neurotransmitter receptor binding and downstream transmission in the postsynaptic cell* (marginal *P* < 10^−13^). Interestingly, these results implicated synaptic function in the behaving organism rather than early brain development.

**Fig. 5:**
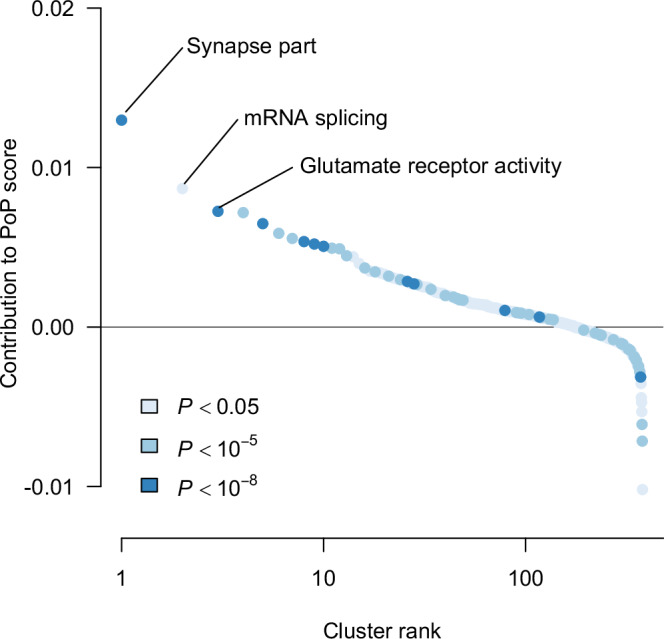
Feature clusters ranked by contribution to the PoP scores of genes. In this analysis we used only DEPICT gene sets [59] as features. Clustering was performed with the affinity-propagation algorithm [83]. Color represents the marginal *P* value of the cluster’s exemplary feature in the univariate prediction of MAGMA gene-prioritization score. Supplementary Table S11 gives the complete results.

The very top-ranked features may have been dominated by the expression data because of its completeness; the database categories were missing more entries. We reran PoPS with a subset of biologically informative features and clustered the top results. Using the sum of the effect sizes of a cluster’s highly correlated members as the overall effect size, we found *synapse part* to be the top cluster (Fig. 5), confirming the importance of synaptic communication. The second-ranked cluster was *mRNA splicing*, perhaps a surprising result. When we used S-LDSC to estimate the heritability enrichment of the related gene sets *RNA splicing* (1.59-fold enrichment, *P* < 0.01) and *mRNA processing* (1.56-fold enrichment, *P* < 0.01), we obtained support for the PoPS result (Supplementary Table S12). We also note that these gene sets were found to be enriched in an earlier GWAS of EA [5]. It is tempting to speculate that different proteins resulting from transcript variants of the same gene may have distinct effects on a given group factor or specific ability.

Several of the PoPS-prioritized genes encode proteins described by NCBI Gene (Supplementary Table S13) as either regulating mRNA splicing (*ZPF64*, *FAM172A*) or possessing multiple transcript variants.

The third-ranked cluster in the PoPS enrichment analysis was *glutamate receptor activity* (Fig. 5). Glutamate is the most abundant excitatory neurotransmitter, and this result confirmed the importance of synaptic function to the quantitative factor. The prioritized gene *GRM8* encodes a glutamate metabotropic receptor, while *NGEF* encodes a guanyl-nucleotide exchange factor predicted to be active in the signaling pathway downstream of metabotropic transmitter-receptor binding.

Other results of the S-LDSC heritability-enrichment analysis also provided support for the inferences presented above. The significantly enriched gene sets included *synapse organization* (1.43-fold enrichment, *P* < 10^−3^), *axon guidance* (1.57-fold enrichment, *P* < 0.005), *regulation of neuron projection development* (1.47-fold enrichment, *P* < 0.005), and *synaptic membrane* (1.37-fold enrichment, *P* < 0.005).

## Discussion

This study marks the first attempted GWAS of quantitative ability as a group factor and the second of any group factor in the domain of mental abilities [18]. Using structural equation modeling, we examined genetic associations with a latent factor indicated by self-reported math ability and highest math class taken. We discovered 53 independent genetic associations with the quantitative factor, 16 of which had not been statistically significant in any previous GWAS of a cognitive trait.

Our study did have a critical limitation. To obtain model identification with a limited number of indicators, we assumed each indicator’s genetic variance to be completely accounted for by the common factors. This assumption led to likely distortions in the relative sizes of the factor loadings. It is easy to recommend broader and more precise phenotyping of behavioral traits in large GWAS to address this kind of shortcoming, but hard to execute in practice for even the small handful of researchers able to influence these decisions. An alternative may be to rely on measures of group factors that many people have already taken in the course of aptitude testing [16, 69] or gamified measures capable of yielding a high throughput [70, 71].

The PGS based on our generated summary statistics was able to predict a small but marginally significant (*P* = 0.01) fraction of the variance in a test of arithmetic achievement. The coefficient of the PGS appeared to be similar (again *P* = 0.01) upon including the parental PGS in the regression model, suggesting a lack of strong confounding bias. The PGS also seemed to show discriminant validity in that its coefficients in the prediction of reading and spelling achievement were near zero.

Genetic influences on the quantitative factor act disproportionately through their effects on the brain, as demonstrated by the enriched tissues and gene sets. By now this result is expected whenever the trait studied in a GWAS is behavioral in nature. What is perhaps unexpected is the near-zero genetic correlation of the quantitative factor with brain size, a trait showing robust associations with intelligence and years of education (Fig. 3). Previous studies have suggested that the correlation between brain size and abilities might be explained primarily as on effect on *g* [72–75], raising an intriguing possibility: while the sheer number of computing units may affect general ability most of all, how the units connect to each other (e.g., *regulation of neuron projection development*) or how their interfaces operate in real time (e.g., *glutamate receptor activity*) may affect both general ability and specialization in certain directions.

The quantitative factor is genetically correlated with higher subjective well-being, less depression, and lower neuroticism (Fig. 3). This finding is consistent with the meta-analytic negative phenotypic correlations of objectively measured quantitative abilities with neuroticism, negative affect, depression, and anxiety [76], although it is likely that an association between well-being and the non-ability components of our quantitative factor (self-confidence, a positive attitude) also plays a role [35, 43, 77–79].

Other noteworthy genetic correlations include not only the expected positive correlations with the programmer, mathematician, and physicist job codes but also the negative correlations with occupations defined by the promulgation of policies and opinions (writer, NGO/union organizer, government official, journalist) (Fig. 3). This pattern agrees with the recently reported negative genetic correlations (controlling for overall EA) between educational specialization in natural sciences, mathematics, and statistics on the one hand and in business, administration, law, social sciences, and journalism on the other [80]. The latter finding might be dismissed as an artifact of specialization in one subject mostly excluding specialization in another, but it is supported by another study reporting that the SAT Verbal positively predicted political participation (see also [81]) even as in the same multiple regressions the SAT Math featured a null or negative coefficient [82].

## Supplementary information

Supplementary Information

Supplementary tables S1-S12

## Data Availability

The key datasets used in the current study—the GWAS summary statistics of self-reported math ability and highest math class taken—are not publicly available and must be requested from 23andMe. The supporting datasets are publicly available, possibly subject to some restrictions, and are described in the Supplementary Information.

## References

[1] Spearman C. The Abilities of Man: Their Nature and Measurement. New York: Macmillan; 1927.

[2] Jensen AR. The g Factor: The Science of Mental Ability. Westport: Praeger; 1998.

[3] Sniekers S, Stringer S, Watanabe K, Jansen PR, Coleman JRI, Krapohl E, et al. Genome-wide association meta-analysis of 78 308 individuals identifies new loci and genes influencing human intelligence. Nat Genet. 2017;49:1107–12. 28530673 10.1038/ng.3869 PMC5665562

[4] Savage JE, Jansen PR, Stringer S, Watanabe K, Bryois J, de Leeuw CA, et al. Genome-wide association meta-analysis in 269 867 individuals identifies new genetic and functional links to intelligence. Nat Genet. 2018;50:912–9. 29942086 10.1038/s41588-018-0152-6 PMC6411041

[5] Lee JJ, Wedow R, Okbay A, Kong E, Maghzian O, Zacher M, et al. Gene discovery and polygenic prediction from a genome-wide association study of educational attainment in 1.1 million individuals. Nat Genet. 2018;50:1112–21. 30038396 10.1038/s41588-018-0147-3 PMC6393768

[6] de la Fuente J, Davies G, Grotzinger AD, Tucker-Drob EM, Deary IJ. A general dimension of genetic sharing across diverse cognitive traits inferred from molecular data. Nat Hum Behav. 2021;5:49–58. 32895543 10.1038/s41562-020-00936-2 PMC9346507

[7] Thurstone LL. Primary Mental Abilities. Chicago: University of Chicago Press; 1938.

[8] Vernon PE. The Structure of Human Abilities. New York: Wiley; 1950.

[9] Horn JL. Human abilities: a review of research and theory in the early 1970s. Annu Rev Psychol. 1976;27:437–85. 773264 10.1146/annurev.ps.27.020176.002253

[10] Cattell RB. Intelligence: Its Structure. Growth and Action. Amsterdam: Elsevier; 1987.

[11] Carroll JB. Human Cognitive Abilities: A Survey of Factor-Analytic Studies. New York: Cambridge University Press; 1993.

[12] Johnson W, Bouchard TJ Jr. The structure of human intelligence: it is verbal, perceptual, and image rotation (VPR), not fluid and crystallized. Intelligence. 2005;33:393–416.

[13] Humphreys LG, Lubinski D, Yao G. Utility of predicting group membership and the role of spatial visualization in becoming an engineer, physical scientist, or artist. J Appl Psychol. 1993;78:250–61. 8482696 10.1037/0021-9010.78.2.250

[14] Gohm CL, Humphreys LG, Yao G. Underachievement among spatially gifted students. Am Educ Res J. 1998;35:515–31.

[15] Shea DL, Lubinski D, Benbow CP. Importance of assessing spatial ability in intellectually talented young adolescent: A 20-year longitudinal study. J Educ Psychol. 2001;93:604–14.

[16] Park G, Lubinski D, Benbow CP. Contrasting intellectual patterns predict creativity in the arts and sciences: Tracking intellectually precocious youth over 25 years. Psychol Sci. 2007;18:948–52. 17958707 10.1111/j.1467-9280.2007.02007.x

[17] Wai J, Lubinski D, Benbow CP. Spatial ability for STEM domains: Aligning over 50 years of cumulative psychological knowledge solidifies its importance. J Educ Psychol. 2009;101:817–35.

[18] Jonsdottir GA, Einarsson GV, Thorleifsson G, Magnusson SH, Gunnarsson AF, Frigge ML, et al. Genetic propensities for verbal and spatial ability have opposite effects on body mass index and risk of schizophrenia. Intelligence. 2021;88:101565.

[19] Wai J, Lee MH, Kell HJ. Distributions of academic math-verbal tilt and overall academic skill of students specializing in different fields: a study of 1.6 million Graduate Record Examination test takers. Intelligence. 2022;95:101701.

[20] Werdelin I The Mathematical Ability: Experimental and Factorial Studies. Gleerup: Lund, 1958.

[21] Keith TZ, Reynolds MR. Cattell-Horn-Carroll abilities and cognitive tests: What we’ve learned from 20 years of research. Psychol Sch. 2010;47:635–50.

[22] Gignac GE. Raven’s is not a pure measure of general intelligence: Implications for *g* factor theory and the brief measurement of *g*. Intelligence. 2015;52:71–9.

[23] Jensen AR. Bias in Mental Testing. New York: Free Press; 1980.

[24] Raven J, Raven JC, Court JH. Manual for Raven’s Progressive Matrices and Vocabulary Scales. San Antonio: Harcourt; 1998.

[25] Pokropek A, Marks GN, Borgonovi F. How much do students’ scores in PISA reflect general intelligence and how much do they reflect specific abilities? J Educ Psychol. 2022;114:1121–35.

[26] Lubinski D, Benbow CP. Study of Mathematically Precocious Youth after 35 years: Uncovering antecedents for the development of math-science expertise. Perspect Psychol Sci. 2006;1:316–45. 26151798 10.1111/j.1745-6916.2006.00019.x

[27] Coyle TR. Non-*g* factors predict educational and occupational criteria: More than *g*. J Intell. 2018;6:43. 31162470 10.3390/jintelligence6030043 PMC6480787

[28] Aucejo E, James J. The path to college education. The role of math and verbal skills. J Politcal Econ. 2021;129:2905–46.

[29] Lubinski D, Humphreys LG. A broadly based analysis of mathematical giftedness. Intelligence. 1990;14:327–55.

[30] Alarcón M, Knopik VS, DeFries JC. Covariation of mathematics achievement and general cognitive ability in twins. J Sch Psychol. 2000;38:63–77.

[31] Kovas Y, Harlaar N, Petrill SA, Plomin R. ‘Generalist genes’ and mathematics in 7-year-old twins. Intelligence. 2005;33:473–89. 19319204 10.1016/j.intell.2005.05.002 PMC2659657

[32] Trzaskowski M, Davis OSP, DeFries JC, Yang J, Visscher PM, Plomin R. DNA evidence for strong genome-wide pleiotropy of cognitive and learning abilities. Behav Genet. 2013;43:267–73. 23609157 10.1007/s10519-013-9594-x PMC3690183

[33] Procopio F, Zhou Q, Wang Z, Gidziela A, Rimfeld K, Malanchini M, et al. The genetics of specific cognitive abilities. Intelligence. 2022;95:101689. 37197611 10.1016/j.intell.2022.101689 PMC10184120

[34] Campbell DP, Hyne SA, Nilsen DL. Manual for the Campbell Interest and Skill Survey: CISS. Minneapolis: National Computer Systems; 1992.

[35] Neubauer AC, Hofer G. Self-estimates of abilities are a better reflection of individuals’ personality traits than of their abilities and are also strong predictors of professional interests. Pers Indiv Differ. 2021;169:109850.

[36] Rolfhus EL, Akcerman PL. Self-report knowledge. At the crossroads of ability, interest, and personality. J Educ Psychol. 1996;88:174–88.

[37] Ackerman PL. A theory of adult intellectual development: process, personality, interests, and knowledge. Intelligence. 1996;22:227–57.

[38] Bernstein BO, Lubinski D, Benbow CP. Psychological constellations assessed at age 13 predict distinct forms of eminence 35 years later. Psychol Sci. 2019;30:444–54. 30694728 10.1177/0956797618822524 PMC6419263

[39] Ackerman PL, Heggestad ED. Intelligence, personality, and interests: evidence for overlapping traits. Psychol Bull. 1997;121:219–45. 9100487 10.1037/0033-2909.121.2.219

[40] Robertson KF, Smeets S, Lubinski D, Benbow CP. Beyond the threshold hypothesis: Even among the gifted and top math/science graduate students, cognitive abilities, vocational interests, and lifestyle preferences matter for career choice, performance, and persistence. Curr Dir Psychol Sci. 2010;19:346–51.

[41] Grtozinger AD, Rhemtulla M, de Vlaming R, Ritchie SJ, Mallard TT, Hill WD, et al. Genomic structural equation modelling provides insights into the multivariate genetic architecture of complex traits. Nat Hum Behav. 2019;3:513–25. 30962613 10.1038/s41562-019-0566-x PMC6520146

[42] Starr A, Riemann R. Common genetic and environmental effects on cognitive ability, conscientiousness, self-perceived abilities, and school performance. Intelligence. 2022;93:101664.

[43] Torppa M, Aro T, Eklund K, Parrila R, Eloranta AK, Ahonen T. Adolescent reading and math skills and self-concept beliefs as predictors of age 20 emotional well-being. Read Writ. 2024;37:2075–99.

[44] Neubauer AC, Pribil A, Wallner A, Hofer G. The self-other knowledge asymmetry in cognitive intelligence, emotional intelligence, and creativity. Heliyon. 2018;4:e01061. 30603696 10.1016/j.heliyon.2018.e01061 PMC6307038

[45] Bulik-Sullivan B, Finucane HK, Anttila V, Gusev A, Day FR, Loh PR, et al. An atlas of genetic correlations across human diseases and traits. Nat Genet. 2015;47:1236–41. 26414676 10.1038/ng.3406 PMC4797329

[46] Lee JJ, McGue M, Iacono WG, Chow CC. The accuracy of LD Score regression as an estimator of confounding and genetic correlations in genome-wide association studies. Genet Epidemiol. 2018;42:783–95. 30251275 10.1002/gepi.22161 PMC6758917

[47] Yang J, Ferreira T, Morris AP, Medland SE, Genetic Investigation of Anthropometric Traits Consortium, Diabetes Genetics Replication and Meta-Analysis Consortium. et al. Conditional and joint multiple-SNP analysis of GWAS summary statistics identifies additional variants influencing complex traits. Nat Genet. 2012;44:369–75. 22426310 10.1038/ng.2213 PMC3593158

[48] Wilson S, Haroian K, Iacono WG, Krueger RF, Lee JJ, Luciano M, et al. Minnesota center for twin and family research. Twin Res Hum Genet. 2019;22:746–52. 31796137 10.1017/thg.2019.107 PMC7056536

[49] Ge T, Chen CY, Ni Y, Feng YCA, Smoller JW. Polygenic prediction via Bayesian regression and continuous shrinkage priors. Nat Commun. 2019;10:1776. 30992449 10.1038/s41467-019-09718-5 PMC6467998

[50] Miller MB, Basu S, Cunningham J, Eskin E, Malone SM, Oetting WS, et al. The Minnesota Center for Twin and Family Research genome-wide association study. Twin Res Hum Genet. 2012;15:767–74. 23363460 10.1017/thg.2012.62 PMC3561927

[51] Fisher RA. Statistical methods in genetics. Heredity. 1952;6:1–12.

[52] Laird LM, Lange C. Family-based designs in the age of large-scale gene-association studies. Nat Rev Genet. 2006;7:385–94. 16619052 10.1038/nrg1839

[53] Lee JJ, Chow CC. The causal meaning of Fisher’s average effect. Genet Res. 2013;95:89–109. 10.1017/S0016672313000074 PMC3779663 23938113

[54] Okbay A, Wu Y, Wang N, Jayashankar H, Bennett M, Nehzati SM, et al. Polygenic prediction of educational attainment within and between families from genome-wide association analyses in 3 million individuals. Nat Genet. 2022;54:437–49. 35361970 10.1038/s41588-022-01016-z PMC9005349

[55] Finucane HK, Bulik-Sullivan B, Gusev A, Trynka G, Reshef Y, Loh PR, et al. Partitioning heritability by functional annotation using genome-wide association summary statistics. Nat Genet. 2015;47:1228–35. 26414678 10.1038/ng.3404 PMC4626285

[56] GTEx Consortium. The Genotype-Tissue Expression (GTEx) pilot analysis: multitissue gene regulation in humans. Science. 2015;348:648–60. 25954001 10.1126/science.1262110 PMC4547484

[57] Finucane HK, Reshef YA, Anttila V, Slowikowski K, Gusev A, Byrnes A, et al. Heritability enrichment of specifically expressed genes identifies disease-relevant tissues and cell types. Nat Genet. 2018;50:621–9. 29632380 10.1038/s41588-018-0081-4 PMC5896795

[58] Weeks EM, Ulirsch JC, Cheng NY, Trippe BL, Fine RS, Miao J, et al. Leveraging polygenic enrichments of gene features to predict genes underlying complex traits and diseases. Nat Genet. 2021;55:1267–76. 10.1038/s41588-023-01443-6 PMC10836580 37443254

[59] Pers TH, Karjalainen J, Chan Y, Westra HJ, Wood AR, Yang J, et al. Biological interpretation of genome-wide association studies using predicted gene functions. Nat Commun. 2015;6:5890. 25597830 10.1038/ncomms6890 PMC4420238

[60] Bulik-Sullivan B, Loh PR, Finucane HK, Ripke S, Yang J, Schizophrenia Working Group of the Psychiatric Genomics Consortium. et al. LD Score regression distinguishes confounding from polygenicity in genome-wide association studies. Nat Genet. 2015;47:291–5. 25642630 10.1038/ng.3211 PMC4495769

[61] Demange PA, Malanchini M, Mallard TT, Biroli P, Cox SR, Grotzinger AD, et al. Investigating the genetic architecture of noncognitive skills using GWAS-by-subtraction. Nat Genet. 2021;53:35–44. 33414549 10.1038/s41588-020-00754-2 PMC7116735

[62] Pokropek A, Marks GN, Borgonovi F, Koc P, Grieff S. General or specific abilities? Evidence from 33 countries participating in the PISA assessments. Intelligence. 2022;92:101653.

[63] Schizophrenia Working Group of the Psychiatric Genomics Consortium. Biological insights from 108 schizophrenia-associated genetic loci. Nature. 2014;511:421–7. 25056061 10.1038/nature13595 PMC4112379

[64] Okbay A, Beauchamp JP, Fontana MA, Lee JJ, Pers TH, Rietveld CA, et al. Genome-wide association study identifies 74 loci associated with educational attainment. Nature. 2016;533:539–42. 27225129 10.1038/nature17671 PMC4883595

[65] Kim Y, Saunders GRB, Giannelis A, Willoughby EA, DeYoung GC, Lee JJ. Genetic and neural bases of the neuroticism general factor. Biol Psychol. 2023;184:108692. 37783279 10.1016/j.biopsycho.2023.108692

[66] Rajagopal VM, Ganna A, Coleman JR, Allegrini A, Voloudakis G, Grove J, et al. Genome-wide association study of school grades identifies genetic overlap between language ability, psychopathology and creativity. Sci Rep. 2023;13:429. 36624241 10.1038/s41598-022-26845-0 PMC9829693

[67] Tonizzi I, Usai MC. Math abilities in autism spectrum disorder: a meta-analysis. Res Dev Disabil. 2023;139:104559. 37329855 10.1016/j.ridd.2023.104559

[68] de Leeuw CA, Mooij JM, Heskes T, Posthuma D. MAGMA: Generalized gene-set analysis of GWAS data. PLoS Comput Biol. 2015;11:e1004219. 25885710 10.1371/journal.pcbi.1004219 PMC4401657

[69] Frey MC, Detterman DK. Scholastic assessment or *g*? The relationship between the Scholastic Aptitude Test and general cognitive ability. Psychol Sci. 2004;15:373–8. 15147489 10.1111/j.0956-7976.2004.00687.x

[70] Malanchini M, Rimfeld K, Gidziela A, Cheesman R, Allegrini AG, Shakeshaft N, et al. Pathfinder: A gamified measure to integrate general cognitive ability into the biological, medical, and behavioural sciences. Mol Psychiatry. 2021;26:7823–37. 34599278 10.1038/s41380-021-01300-0 PMC8872983

[71] Landers RN, Armstrong MB, Collmus AB, Mujcic S, Blaik J. Theory-driven game-based assessment of general cognitive ability: design theory, measurement, prediction of performance, and test fairness. J Appl Psychol. 2022;107:1655–77. 34672652 10.1037/apl0000954

[72] Keivit RA, van Rooijen H, Wicherts JM, Waldorp LJ, Kan KJ, Scholte HS, et al. Intelligence and the brain: a model-based approach. Cogn Neurosci. 2012;3:89–97. 24168689 10.1080/17588928.2011.628383

[73] Lee JJ, McGue M, Iacono WG, Michael AM, Chabris CF. The causal influence of brain size on human intelligence: Evidence from within-family phenotypic associations and GWAS modeling. Intelligence. 2019;75:48–58. 32831433 10.1016/j.intell.2019.01.011 PMC7440690

[74] Cox SR, Ritchie SJ, Fawns-Ritchie C, Tucker-Drob EM, Deary IJ. Structural brain imaging correlates of general intelligence in UK Biobank. Intelligence. 2019;76:101376. 31787788 10.1016/j.intell.2019.101376 PMC6876667

[75] Pietschnig J, Gerdesmann D, Zeiler M, Voracek M. Of differing methods, disputed estimates and discordant interpretations: the meta-analytical multiverse of brain volume and IQ associations. R Soc Open Sci. 2022;9:211621. 35573038 10.1098/rsos.211621 PMC9096623

[76] Stanek KC, Ones DS. Meta-analytic relations between personality and cognitive ability. Proc Natl Acad Sci USA. 2023;120:e2212794120. 37252971 10.1073/pnas.2212794120 PMC10266031

[77] Vedel A. Big Five personality group differences across academic majors: a systematic review. Pers Indiv Differ. 2016;92:1–10.

[78] Zajenkowski M. How do teenagers perceive their intelligence? Narcissism, intellect, well-being and gender as correlates of self-assessed intelligence among adolescents. Pers Indiv Differ. 2021;169:109978.

[79] Coenen J, Borghans L, Diris R. Personality traits, preferences and educational choices: a focus on STEM. J Econ Psychol. 2021;84:102361.

[80] Cheesman R, Anapaz V, Ebeltoft JC, Porneso R, Ayorech Z, Demange P, et al. Genetic associations with educational fields in >460,000 individuals. Nat Genet. [in press]. 10.1038/s41588-025-02391-z PMC12695639 41188532

[81] Edwards T, Dawes CT, Willoughby EA, McGue M, Lee JJ. More than *g*: Verbal and performance IQ as predictors of socio-political attitudes. Intelligence. 2025;108:101876. 10.1016/j.intell.2024.101876 PMC12716881 41424531

[82] Hillygus DS. The missing link: Exploring the relationship between higher education and political engagement. Polit Behav. 2005;27:25–47.

[83] Frey BJ, Dueck D. Clustering by passing messages between data points. Science. 2007;315:972–6. 17218491 10.1126/science.1136800

