# Supplementary material for: A genetic common factor underlying self-reported math ability and highest math class taken
Source: Mol Psychiatry. 2025 Sep 20;30(12):5701–9. doi: 10.1038/s41380-025-03237-0 (PMC12602337; doi:10.1038/s41380-025-03237-0)
Supplement: Supplementary file 1 — Supplementary Information [file 41380_2025_3237_MOESM1_ESM.pdf]

Supplementary Information
A genetic common factor underlying self-reported math ability and highest math class taken

Alexandros Giannelis PhD[1,2], Emily A. Willoughby PhD[2], Tobias Edwards MSc[2], Matt McGue PhD[2], James J. Lee PhD[2*]

[1]Department of Applied Economics
Erasmus School of Economics
Erasmus University Rotterdam
Rotterdam, The Netherlands

[2]Department of Psychology
University of Minnesota Twin Cities
75 East River Road
Minneapolis, MN 55455, USA

*e-mail: leex2293@umn.edu; phone: (612) 625-4980

Table S13: List of 48 genes nearest to lead SNPs with summaries of their functions. Summaries taken from the NIH National Library of Medicine–National Center for Biotechnology Information. If "NA", no summary was available for that gene in the NCBI database.

Ensembl ID	Entrez ID	HGNC symbol	Full name	Summary
ENSG00000008197	83741	*TFAP2D*	transcription factor AP-2 delta	Predicted to enable DNA-binding transcription factor activity, RNA polymerase II-specific and RNA polymerase II transcription regulatory region sequence-specific DNA binding activity. Predicted to be involved in anatomical structure development; regulation of cell population proliferation; and regulation of transcription by RNA polymerase II. Predicted to act upstream of or within inferior colliculus development; negative regulation of neuron apoptotic process; and positive regulation of transcription by RNA polymerase II. Predicted to be part of chromatin. Predicted to be active in nucleus.
ENSG00000020256	55734	*ZFP64*	ZFP64 zinc finger protein	Predicted to enable DNA binding activity and metal ion binding activity. Predicted to be involved in positive regulation of cytokine production and positive regulation of transcription by RNA polymerase II. Predicted to act upstream of or within positive regulation of mRNA splicing, via spliceosome. Predicted to be active in nucleus.
ENSG00000049759	23327	*NEDD4L*	NEDD4 like E3 ubiquitin protein ligase	This gene encodes a member of the Nedd4 family of HECT domain E3 ubiquitin ligases. HECT domain E3 ubiquitin ligases transfer ubiquitin from E2 ubiquitin-conjugating enzymes to protein substrates, thus targeting specific proteins for lysosomal degradation. The encoded protein mediates the ubiquitination of multiple target substrates and plays a critical role in epithelial sodium transport by regulating the cell surface expression of the epithelial sodium channel, ENaC. Single nucleotide polymorphisms in this gene may be associated with essential hypertension. Alternatively spliced transcript variants encoding multiple isoforms have been observed for this gene.
ENSG00000066248	25791	*NGEF*	neuronal guanine nucleotide exchange factor	Enables guanyl-nucleotide exchange factor activity. Predicted to be involved in several processes, including activation of GTPase activity; ephrin receptor signaling pathway; and negative regulation of dendritic spine morphogenesis. Predicted to be located in cytosol. Predicted to be active in glutamatergic synapse.
ENSG00000078687	57690	*TNRC6C*	trinucleotide repeat containing adaptor 6C	Predicted to enable RNA binding activity. Involved in gene silencing by miRNA; positive regulation of nuclear-transcribed mRNA catabolic process, deadenylation-dependent decay; and positive regulation of nuclear-transcribed mRNA poly(A) tail shortening. Predicted to be located in cytosol. Predicted to be active in P-body and nucleoplasm.
ENSG00000078725	1620	*BRINP1*	BMP/retinoic acid inducible neural specific 1	This gene is located within a chromosomal region that shows loss of heterozygosity in some bladder cancers. It contains a 5' CpG island that may be a frequent target of hypermethylation, and it may undergo hypermethylation-based silencing in some bladder cancers.

Ensembl ID	Entrez ID	HGNC symbol	Full name	Summary
ENSG00000085365	9522	*SCAMP1*	secretory carrier membrane protein 1	This gene product belongs to the SCAMP family of proteins, which are secretory carrier membrane proteins. They function as carriers to the cell surface in post-golgi recycling pathways. Different family members are highly related products of distinct genes, and are usually expressed together. These findings suggest that these protein family members may function at the same site during vesicular transport rather than in separate pathways. A pseudogene of this gene has been defined on chromosome 1. Alternative splicing results in multiple transcript variants.
ENSG00000099800	26517	*TIMM13*	translocase of inner mitochondrial membrane 13	This gene encodes a member of the evolutionarily conserved TIMM (translocase of inner mitochondrial membrane) family of proteins that function as chaperones in the import of proteins from the cytoplasm into the mitochondrial inner membrane. Proteins of this family play a role in collecting substrate proteins from the translocase of the outer mitochondrial membrane (TOM) complex and delivering them to either the sorting and assembly machinery in the outer mitochondrial membrane (SAM) complex or the TIMM22 complex in the inner mitochondrial membrane. The encoded protein and the translocase of mitochondrial inner membrane 8a protein form a 70 kDa complex in the intermembrane space.
ENSG00000099901	5902	*RANBP1*	RAN binding protein 1	This gene encodes a protein that forms a complex with Ras-related nuclear protein (Ran) and metabolizes guanoside triphosphate (GTP). This complex participates in the regulation of the cell cycle by controlling transport of proteins and nucleic acids into the nucleus. There are multiple pseudogenes for this gene on chromosomes 9, 12, 17, and X. Alternative splicing results in multiple transcript variants.
ENSG00000100393	2033	*EP300*	E1A binding protein p300	This gene encodes the adenovirus E1A-associated cellular p300 transcriptional co-activator protein. It functions as histone acetyltransferase that regulates transcription via chromatin remodeling and is important in the processes of cell proliferation and differentiation. It mediates cAMP-gene regulation by binding specifically to phosphorylated CREB protein. This gene has also been identified as a co-activator of HIF1A (hypoxia-inducible factor 1 alpha), and thus plays a role in the stimulation of hypoxia-induced genes such as VEGF. Defects in this gene are a cause of Rubinstein-Taybi syndrome and may also play a role in epithelial cancer.
ENSG00000101203	57642	*COL20A1*	collagen type XX alpha 1 chain	Predicted to be located in endoplasmic reticulum lumen and extracellular region. Predicted to be part of collagen trimer. Predicted to be active in collagen-containing extracellular matrix and extracellular space.

Ensembl ID	Entrez ID	HGNC symbol	Full name	Summary
ENSG00000102984	55565	*ZNF821*	zinc finger protein 821	This gene encodes a protein with two C2H2 zinc finger motifs and a score-and-three (23)-amino acid peptide repeat (STPR) domain. The STPR domain of the encoded protein binds to double stranded DNA and may also contain a nuclear localization signal, suggesting that this protein interacts with chromosomal DNA. Alternatively spliced transcript variants encoding multiple isoforms have been observed for this gene.
ENSG00000104112	29106	*SCG3*	secretogranin III	The protein encoded by this gene is a member of the chromogranin/secretogranin family of neuroendocrine secretory proteins. Granins may serve as precursors for biologically active peptides. Some granins have been shown to function as helper proteins in sorting and proteolytic processing of prohormones; however, the function of this protein is unknown. Two transcript variants encoding different isoforms have been found for this gene.
ENSG00000105778	23080	*AVL9*	AVL9 cell migration associated	Involved in cell migration. Located in recycling endosome.
ENSG00000108551	51655	*RASD1*	ras related dexamethasone induced 1	This gene encodes a member of the Ras superfamily of small GTPases and is induced by dexamethasone. The encoded protein is an activator of G-protein signaling and acts as a direct nucleotide exchange factor for Gi-Go proteins. This protein interacts with the neuronal nitric oxide adaptor protein CAPON, and a nuclear adaptor protein FE65, which interacts with the Alzheimer's disease amyloid precursor protein. This gene may play a role in dexamethasone-induced alterations in cell morphology, growth and cell-extracellular matrix interactions. Epigenetic inactivation of this gene is closely correlated with resistance to dexamethasone in multiple myeloma cells. Alternatively spliced transcript variants encoding different isoforms have been found for this gene.
ENSG00000113391	83989	*FAM172A*	ARB2 cotranscriptional regulator A	Predicted to contribute to siRNA binding activity. Predicted to be involved in heterochromatin assembly by small RNA; neural crest cell development; and regulation of alternative mRNA splicing, via spliceosome. Located in endoplasmic reticulum.
ENSG00000115392	55120	*FANCL*	FA complementation group L	This gene encodes a ubiquitin ligase that is a member of the Fanconi anemia complementation group (FANC). Members of this group are related by their assembly into a common nuclear protein complex rather than by sequence similarity. This gene encodes the protein for complementation group L that mediates monoubiquitination of FANCD2 as well as FANCI. Fanconi anemia is a genetically heterogeneous recessive disorder characterized by cytogenetic instability, hypersensitivity to DNA crosslinking agents, increased chromosomal breakage, and defective DNA repair. Alternative splicing results in multiple transcript variants.

Ensembl ID	Entrez ID	HGNC symbol	Full name	Summary
ENSG00000118007	10274	*STAG1*	STAG1 cohesin complex component	This gene is a member of the SCC3 family and is expressed in the nucleus. It encodes a component of cohesin, a multisubunit protein complex that provides sister chromatid cohesion along the length of a chromosome from DNA replication through prophase and prometaphase, after which it is dissociated in preparation for segregation during anaphase.
ENSG00000118596	9194	*SLC16A7*	solute carrier family 16 member 7	This gene is a member of the monocarboxylate transporter family. Members in this family transport metabolites, such as lactate, pyruvate, and ketone bodies. The protein encoded by this gene catalyzes the proton-linked transport of monocarboxylates and has the highest affinity for pyruvate. This protein has been reported to be more highly expressed in prostate and colorectal cancer specimens when compared to control specimens. Alternative splicing results in multiple transcript variants.
ENSG00000119862	29094	*LGALSL*	galectin like	Predicted to enable carbohydrate binding activity.
ENSG00000122756	1271	*CNTFR*	ciliary neurotrophic factor receptor	This gene encodes a member of the type 1 cytokine receptor family. The encoded protein is the ligand-specific component of a tripartite receptor for ciliary neurotrophic factor, which plays a critical role in neuronal cell survival, differentiation and gene expression. Binding of ciliary neurotrophic factor to the encoded protein recruits the transmembrane components of the receptor, gp130 and leukemia inhibitory factor receptor, facilitating signal transduction. Single nucleotide polymorphisms in this gene may be associated with variations in muscle strength, as well as early onset of eating disorders. Alternatively spliced transcript variants have been observed for this gene.
ENSG00000130287	1463	*NCAN*	neurocan	Neurocan is a chondroitin sulfate proteoglycan thought to be involved in the modulation of cell adhesion and migration.
ENSG00000134508	91768	*CABLES1*	Cdk5 and Abl enzyme substrate 1	This gene encodes a protein involved in regulation of the cell cycle through interactions with several cyclin-dependent kinases. One study (PMID: 16177568) reported aberrant splicing of transcripts from this gene which results in removal of the cyclin binding domain only in human cancer cells, and reduction in gene expression was shown in colorectal cancers (PMID: 17982127). Multiple transcript variants encoding different isoforms have been found for this gene.

Ensembl ID	Entrez ID	HGNC symbol	Full name	Summary
ENSG00000134769	1837	*DTNA*	dystrobrevin alpha	The protein encoded by this gene belongs to the dystrobrevin subfamily of the dystrophin family. This protein is a component of the dystrophin-associated protein complex (DPC), which consists of dystrophin and several integral and peripheral membrane proteins, including dystroglycans, sarcoglycans, syntrophins and alpha- and beta-dystrobrevin. The DPC localizes to the sarcolemma and its disruption is associated with various forms of muscular dystrophy. Mutations in this gene are associated with left ventricular noncompaction with congenital heart defects. Multiple alternatively spliced transcript variants encoding different isoforms have been identified for this gene.
ENSG00000134802	29015	*SLC43A3*	solute carrier family 43 member 3	Predicted to enable transmembrane transporter activity. Predicted to be involved in transmembrane transport. Predicted to be integral component of membrane.
ENSG00000135916	81618	*ITM2C*	integral membrane protein 2C	Enables amyloid-beta binding activity. Involved in negative regulation of neuron projection development and neuron differentiation. Located in several cellular components, including Golgi apparatus; lysosome; and perinuclear region of cytoplasm.
ENSG00000137872	80031	*SEMA6D*	semaphorin 6D	Semaphorins are a large family, including both secreted and membrane associated proteins, many of which have been implicated as inhibitors or chemorepellents in axon pathfinding, fasciculation and branching, and target selection. All semaphorins possess a semaphorin (Sema) domain and a PSI domain (found in plexins, semaphorins and integrins) in the N-terminal extracellular portion. Additional sequence motifs C-terminal to the semaphorin domain allow classification into distinct subfamilies. Results demonstrate that transmembrane semaphorins, like the secreted ones, can act as repulsive axon guidance cues. This gene encodes a class 6 vertebrate transmembrane semaphorin that demonstrates alternative splicing. Several transcript variants have been identified and expression of the distinct encoded isoforms is thought to be regulated in a tissue- and development-dependent manner.
ENSG00000138821	64116	*SLC39A8*	solute carrier family 39 member 8	This gene encodes a member of the SLC39 family of solute-carrier genes, which show structural characteristics of zinc transporters. The encoded protein is glycosylated and found in the plasma membrane and mitochondria, and functions in the cellular import of zinc at the onset of inflammation. It is also thought to be the primary transporter of the toxic cation cadmium, which is found in cigarette smoke. Multiple transcript variants encoding different isoforms have been found for this gene. Additional alternatively spliced transcript variants of this gene have been described, but their full-length nature is not known.

Ensembl ID	Entrez ID	HGNC symbol	Full name	Summary
ENSG00000140945	1012	*CDH13*	cadherin 13	This gene encodes a member of the cadherin superfamily. The encoded protein is localized to the surface of the cell membrane and is anchored by a GPI moiety, rather than by a transmembrane domain. The protein lacks the cytoplasmic domain characteristic of other cadherins, and so is not thought to be a cell-cell adhesion glycoprotein. This protein acts as a negative regulator of axon growth during neural differentiation. It also protects vascular endothelial cells from apoptosis due to oxidative stress, and is associated with resistance to atherosclerosis. The gene is hypermethylated in many types of cancer. Alternative splicing results in multiple transcript variants encoding different isoforms.
ENSG00000141279	9520	*NPEPPS*	aminopeptidase puromycin sensitive	This gene encodes the puromycin-sensitive aminopeptidase, a zinc metallopeptidase which hydrolyzes amino acids from the N-terminus of its substrate. The protein has been localized to both the cytoplasm and to cellular membranes. This enzyme degrades enkaphalins in the brain, and studies in mouse suggest that it is involved in proteolytic events regulating the cell cycle.
ENSG00000143614	57459	*GATAD2B*	GATA zinc finger domain containing 2B	This gene encodes a zinc finger protein transcriptional repressor. The encoded protein is part of the methyl-CpG-binding protein-1 complex, which represses gene expression by deacetylating methylated nucleosomes. Mutations in this gene are linked to intellectual disability and dysmorphic features associated with cognitive disability.
ENSG00000143776	8476	*CDC42BPA*	CDC42 binding protein kinase alpha	The protein encoded by this gene is a member of the serine/threonine protein kinase family. This kinase contains multiple functional domains. Its kinase domain is highly similar to that of the myotonic dystrophy protein kinase (DMPK). This kinase also contains a Rac interactive binding (CRIB) domain, and has been shown to bind CDC42. It may function as a CDC42 downstream effector mediating CDC42 induced peripheral actin formation, and promoting cytoskeletal reorganization. Multiple alternatively spliced transcript variants have been described.
ENSG00000143919	79823	*CAMKMT*	calmodulin-lysine N-methyltransferase	This gene encodes a class I protein methyltransferase that acts in the formation of trimethyllysine in calmodulin. The protein contains a AdoMet-binding motif and may play a role in calcium-dependent signaling.
ENSG00000145934	57451	*TENM2*	teneurin transmembrane protein 2	Enables cell adhesion molecule binding activity and signaling receptor binding activity. Involved in several processes, including calcium-mediated signaling using intracellular calcium source; heterophilic cell-cell adhesion via plasma membrane cell adhesion molecules; and retrograde trans-synaptic signaling by trans-synaptic protein complex. Located in cell-cell junction and plasma membrane.

Ensembl ID	Entrez ID	HGNC symbol	Full name	Summary
ENSG00000153253	6328	*SCN3A*	sodium voltage-gated channel alpha subunit 3	Voltage-gated sodium channels are transmembrane glycoprotein complexes composed of a large alpha subunit with 24 transmembrane domains and one or more regulatory beta subunits. They are responsible for the generation and propagation of action potentials in neurons and muscle. This gene encodes one member of the sodium channel alpha subunit gene family, and is found in a cluster of five alpha subunit genes on chromosome 2. Multiple transcript variants encoding different isoforms have been found for this gene.
ENSG00000171044	286046	*XKR6*	XK related 6	Predicted to be involved in apoptotic process involved in development; engulfment of apoptotic cell; and phosphatidylserine exposure on apoptotic cell surface. Predicted to be integral component of membrane. Predicted to be active in plasma membrane.
ENSG00000172260	257194	*NEGR1*	neuronal growth regulator 1	Predicted to act upstream of or within several processes, including feeding behavior; locomotory behavior; and positive regulation of neuron projection development. Predicted to be located in extracellular region and plasma membrane.
ENSG00000175182	131408	*FAM131A*	family with sequence similarity 131 member A	NA
ENSG00000175745	7025	*NR2F1*	nuclear receptor subfamily 2 group F member 1	The protein encoded by this gene is a nuclear hormone receptor and transcriptional regulator. The encoded protein acts as a homodimer and binds to 5'-AGGTCA-3' repeats. Defects in this gene are a cause of Bosch-Boonstra optic atrophy syndrome (BBOAS).
ENSG00000178074	205327	*C2orf69*	chromosome 2 open reading frame 69	Involved in oxidative phosphorylation. Located in mitochondrion.
ENSG00000179603	2918	*GRM8*	glutamate metabotropic receptor 8	L-glutamate is the major excitatory neurotransmitter in the central nervous system and activates both ionotropic and metabotropic glutamate receptors. Glutamatergic neurotransmission is involved in most aspects of normal brain function and can be perturbed in many neuropathologic conditions. The metabotropic glutamate receptors are a family of G protein-coupled receptors, that have been divided into 3 groups on the basis of sequence homology, putative signal transduction mechanisms, and pharmacologic properties. Group I includes GRM1 and GRM5 and these receptors have been shown to activate phospholipase C. Group II includes GRM2 and GRM3 while Group III includes GRM4, GRM6, GRM7 and GRM8. Group II and III receptors are linked to the inhibition of the cyclic AMP cascade but differ in their agonist selectivities. Alternatively spliced transcript variants encoding different isoforms have been described for this gene.

Ensembl ID	Entrez ID	HGNC symbol	Full name	Summary
ENSG00000182050	25834	*MGAT4C*	MGAT4 family member C	Predicted to enable acetylglucosaminyltransferase activity. Predicted to be involved in protein N-linked glycosylation. Predicted to be located in Golgi membrane.
ENSG00000182732	9628	*RGS6*	regulator of G protein signaling 6	This gene encodes a member of the RGS (regulator of G protein signaling) family of proteins, which are defined by the presence of a RGS domain that confers the GTPase-activating activity of these proteins toward certain G alpha subunits. This protein also belongs to a subfamily of RGS proteins characterized by the presence of DEP and GGL domains, the latter a G beta 5-interacting domain. The RGS proteins negatively regulate G protein signaling, and may modulate neuronal, cardiovascular, lymphocytic activities, and cancer risk. Many alternatively spliced transcript variants encoding different isoforms with long or short N-terminal domains, complete or incomplete GGL domains, and distinct C-terminal domains, have been described for this gene, however, the full-length nature of some of these variants is not known.
ENSG00000184349	1946	*EFNA5*	ephrin A5	Ephrin-A5, a member of the ephrin gene family, prevents axon bundling in cocultures of cortical neurons with astrocytes, a model of late stage nervous system development and differentiation. The EPH and EPH-related receptors comprise the largest subfamily of receptor protein-tyrosine kinases and have been implicated in mediating developmental events, particularly in the nervous system. EPH receptors typically have a single kinase domain and an extracellular region containing a Cys-rich domain and 2 fibronectin type III repeats. The ephrin ligands and receptors have been named by the Eph Nomenclature Committee (1997). Based on their structures and sequence relationships, ephrins are divided into the ephrin-A (EFNA) class, which are anchored to the membrane by a glycosylphosphatidylinositol linkage, and the ephrin-B (EFNB) class, which are transmembrane proteins. The Eph family of receptors are similarly divided into 2 groups based on the similarity of their extracellular domain sequences and their affinities for binding ephrin-A and ephrin-B ligands.

Ensembl ID	Entrez ID	HGNC symbol	Full name	Summary
ENSG00000187164	57698	*SHTN1*	shootin 1	Enables identical protein binding activity. Involved in positive regulation of neuron migration. Located in cytoplasm.
ENSG00000188107	346007	*EYS*	eyes shut homolog	The product of this gene contains multiple epidermal growth factor (EGF)-like and LamG domains. The protein is expressed in the photoreceptor layer of the retina, and the gene is mutated in autosomal recessive retinitis pigmentosa. Multiple transcript variants encoding different isoforms have been found for this gene.
ENSG00000204335	389058	*SP5*	Sp5 transcription factor	Predicted to enable DNA-binding transcription factor activity, RNA polymerase II-specific and RNA polymerase II cis-regulatory region sequence-specific DNA binding activity. Predicted to be involved in regulation of transcription by RNA polymerase II. Predicted to act upstream of or within bone morphogenesis; cellular response to organic cyclic compound; and post-anal tail morphogenesis. Predicted to be located in nucleus. Predicted to be part of chromatin.
ENSG00000213722	23564	*DDAH2*	DDAH family member 2, ADMA-independent	This gene encodes a dimethylarginine dimethylaminohydrolase. The encoded enzyme functions in nitric oxide generation by regulating the cellular concentrations of methylarginines, which in turn inhibit nitric oxide synthase activity. The protein may be localized to the mitochondria. Alternative splicing resulting in multiple transcript variants.

Additional Materials and Methods

All statistical tests performed specifically for this study, other than the Q_{SNP} tests, were two sided.

Observed phenotypes

The GWAS of self-reported math ability ($N = 564\,698$) and highest math class taken ($N = 430\,445$) were conducted exclusively among research participants of 23andMe, Inc. who answered survey questions about their mathematical background. Lead SNPs identified in these GWAS were presented in a previous paper [1], but without further analysis it is unclear whether these SNPs are tagging causal sites affecting the quantitative factor specifically. The summary statistics of these GWAS are not publicly available and must be requested from 23andMe.

Self-reported math ability was assessed with the item: *How would you rate your mathematical ability?* Subjects responded to a 5-point scale ranging from *very poor* (1) to *excellent* (5). The mean of this item was 4.71, indicating a ceiling effect in the 23andMe sample. The standard deviation was 0.99. A study employing an extended twin design found that self-reported math ability is at least moderately heritable and correlated with objective ability measurements and

grades in math ($r_g > 0.7$) [2]. Another study found a substantial phenotypic correlation ($r > 0.6$) between self-reported math ability and an objective achievement test [3]. Self-reported quantitative ability appears to be considerably more correlated with measured quantitative ability than are other self-reported abilities with their corresponding measured abilities [4, 5]. These latter studies reported phenotypic correlations between self-reported numerical ability and scores on objective tests of number-series completion ranging from 0.41 and 0.53. The verbal and spatial correlations between self-report and objective scores ranged from 0.08 to 0.31.

Highest math class taken was assessed with the item: *Excluding statistics courses, what is the most advanced math class you have successfully completed?* Subjects responded to an 8-point scale: *pre-algebra* (1), *algebra* (2), *geometry* (3), *trigonometry* (4), *pre-calculus* (5), *calculus* (6), *vector calculus* (7), *more than vector calculus* (8). This item could be faulted for ambiguity, as in the United States algebra is often spread out over at least two non-consecutive year-long courses. Trigonometry and pre-calculus are not always taught in this strict sequence. Nevertheless the mean of this item, 3.44, indicating a stopping point between geometry and trigonometry, was roughly consistent with a recent study reporting that 78 percent of American high school students complete geometry, 34 percent pre-calculus, and 19 percent calculus [6]. The standard deviation of this item was a considerable 3.82.

Our structural equation modeling also employed GWAS summary statistics for cognitive performance (CP) and educational attainment (EA). For most of our analyses, we used a version of the CP summary statistics generated by the Social Science Genetic Association Consortium (SSGAC) ($N = 257\,841$) [1]. To generate summary statistics of the quantitative factor for purposes of polygenic prediction, we used a distinct set of summary statistics to represent CP because the SSGAC meta-analysis included the contribution of the Minnesota Center for Twin and Family Research (MCTFR). Note that MCTFR was the validation sample available to us for polygenic prediction. In the place of the SSGAC summary statistics, we used those of the g-factor summary statistics generated by de la Fuente et al. [7] exclusively from the UK Biobank.

We used a version of the EA summary statistics generated by the SSGAC [1] excluding MCTFR and 23andMe ($N = 762\,524$). Note that the MCTFR contribution to the SSGAC meta-analysis of CP was part of a prior meta-analysis that was carried forward, and contributions to this prior meta-analysis cannot easily be separated out.

Methodological details of the original GWAS of these phenotypes were reported previously [1] and are partially repeated here. The EA phenotype was constructed in its original GWAS by mapping each major educational qualification that can be identified from the survey measure of the cohort to an International Standard Classification of Education (ISCED) category and imputing a years-of-education equivalent for each ISCED category. CP was measured in the UKB with a 13-item test of verbal-numerical reasoning administered with a two-minute time

limit. A given UKB participant took this test up to four times, and the mean of all occasions was used as the phenotype.

All cohort-level analyses of EA were restricted to individuals of European ancestry who passed the quality control of the cohort and for whom EA was measured at age 30 or older. In each cohort-level GWAS of EA, the analyst was asked to control for the first 10 principal components (PCs) of the genetic data after the removal of genetic outliers (20 PCs were used in the analysis of the UKB) and additional covariates that included the first three powers of birth year, an indicator for being female, and their interactions. Supplementary Table 19 of this earlier paper gave the details of any deviations from the protocol by a given cohort. The meta-analysts applied a myriad of additional quality-control checks to each cohort file. The software program METAL [8] was used to perform sample-size-weighted meta-analyses of all SNPs passing the quality-control criteria, and the resulting standard errors were inflated by the square root of the intercept of LD Score regression (LDSC) [9, 10].

The single-cohort GWAS of CP (in the UKB only), self-reported math ability, and highest math class taken were conducted identically except with respect to the age criteria. Only 23andMe respondents aged 25 and older at the time of survey were included in the analysis of highest math class taken, and no age filter was applied to CP. UKB participants tended to be somewhat older, typically being between 45 and 70 at the time of assessment.

The sample sizes of these four GWAS were not determined by a power analysis or any predetermined criterion. Each major GWAS of EA, following the first [11], strove to make a substantial improvement upon its predecessor, leading consistently to a roughly threefold increase in sample size each time [1, 12, 13]. The GWAS of CP, self-reported math ability, and highest math class taken accompanying the third major GWAS of EA simply used the largest sample sizes available at the time.

We conducted no new GWAS for this paper and so did not obtain approval from a Research Ethics Committee or Institutional Review Board for that component. The following statements from the Supplementary Information of Lee et al. [1] described the approvals and informed consent pertinent to the archived data that we employed:

- “The lead PI of each cohort affirmed that the results contributed to the study [GWAS meta-analysis of educational attainment] were based on analyses approved by the local Research Ethics Committee and/or Institutional Review Board responsible for overseeing research.” (p. 6)

- “23andMe research participants provided informed consent to take part in this research [on self-reported math ability, highest math class taken, and educational attainment] under a protocol approved by the AAHRPP-accredited institutional review board, Ethical and Independent Review Services. We would like to thank the research participants and

employees of 23andMe for making this work possible.” (p. 139)

- “This research [on cognitive performance and educational attainment] has also been conducted using the UK Biobank resource under Application Numbers 11425 and 12514. Informed consent was obtained from UK Biobank subjects.” (p. 145)

Confirmatory factor analysis and latent-level GWAS

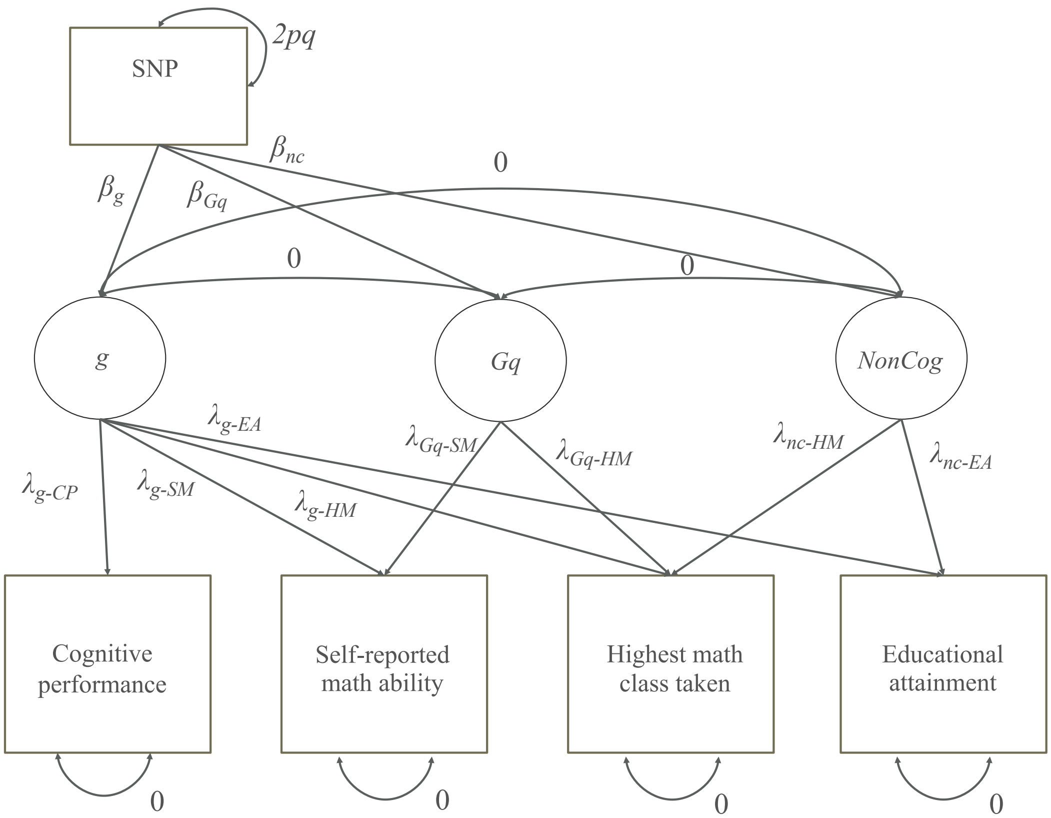

Figure S1: Genomic SEM model with SNP effects.

We used the software tool Genomic SEM [14] to calculate the genetic covariance matrix of the phenotypes studied in the GWAS described above. For this purpose Genomic SEM calls bivariate LDSC [10, 15]. Standard procedures were followed (e.g., including only HapMap3 SNPs with minor allele frequency > 0.01). The standard error of each quantity calculated by LDSC (heritabilities, intercepts, genetic correlations, enrichments) were calculated with the block jackknife [9, 15–17]. This method becomes conservative at very large sample sizes because of inherent differences between blocks of the genome that do not vanish with sample size,

as seen in the very small reduction in the standard errors of enrichments from Lee et al. [1] to Okbay et al. [13].

A factor-analytic model was based on the genetic covariance matrix. Since our model contains four observed indicators and three common factors, it is not identified without further constraints. We chose as constraints the setting of all indicator residual variances to zero. Another way to state this is that we treated each indicator's genetic variance as completely accounted for by one or more of the common factors. This identification strategy should not be wholly unreasonable if g and a prominent group factor typically account for the great bulk of an indicator's heritable variation.

A GWAS of the quantitative factor, measured by self-reported math ability and highest math class taken, was conducted with Genomic SEM (Supplementary Fig. S1). We used diagonal weighted least squares as the estimation method and unit-loading identification. Cognitive performance was the reference indicator of g; self-reported math ability was the reference indicator of the quantitative factor; educational attainment was the reference indicator of *NonCog*. We used the reference file supplied by Genomic SEM to retain only SNPs with minor allele frequency (MAF) > 0.005 in the 1000 Genomes European populations. This left nearly 6 million SNPs in the GWAS. Genomic SEM may not give accurate P values for rare SNPs (B. Verhulst, personal communication), but the Manhattan plot (Fig. 2) shows an absence of free-floating hits without "LD support," which would be likely candidates for this problem.

Unit-loading identification implies that any SNP with an effect of β on the quantitative factor has the same effect β on self-reported math ability. Since the respective standardized loadings of this indicator on g and the quantitative factor were estimated to be 0.644 and 0.764, it follows that about 58 percent of the indicator's genetic variance was attributed to the quantitative factor. Self-reported math ability was estimated by LDSC to have a common-SNP heritability of 0.16 (Table 1), meaning that the heritability of this indicator contributed by the quantitative factor must be $0.16 \times 0.58 \approx 0.09$. Therefore, all common SNPs across the genome affecting the quantitative factor are constrained to satisfy the relation

$$0.09 \approx \sum 2 \times \mathrm{MAF} \times (1 - \mathrm{MAF}) \times \beta^2.$$

This quantity will equal the naive common-SNP heritability in a typical non-latent GWAS, meaning roughly 0.10 if the phenotype is years of education or 0.20 if it is cognitive performance (Table 1). This explanation of the scaling should be used to judge the sizes of the GWAS regression coefficients given in our study (Supplementary Table S2).

The effective sample size of the latent-level GWAS was estimated using a previously suggested method [18] to be $N_e = 343\,237$.

Upon completion of the GWAS, the summary statistics were clumped with PLINK 1.9 [19]. We used a primary P-value threshold of 5×10^{-8}—the standard multiple-correction threshold

used in GWAS—and a secondary P-value threshold of 1×10^{-4}. SNPs were assigned to clumps according to a r^2 threshold of 0.1 and a 1-Mb window. By setting a wide window, we in effect only considered LD for assessing independence between SNPs. LD was calculated using the 1000 Genomes phase 3 European participants as a reference panel [20]. This procedure formed 78 clumps from 5 203 genome-wide significant variants.

These 78 SNPs were then examined using GCTA-COJO [21], to identify conditionally and jointly associated SNPs. Following the developers' recommendation, we did not use 1000 Genomes as the LD reference panel for the COJO analysis. Instead, we used the parents in the MCTFR cohort [22]. The COJO analysis identified 53 SNPs, which we consider to be the main GWAS hits.

The COJO SNPs were then tested for a significant Q_{SNP} statistic. The definition of Q_{SNP} is the difference in χ^2 between a model specifying a path from the SNP to the common factor and a less restrictive model specifying paths to the indicators of that factor. In our case, under the null hypothesis that the more parsimonious model (mediation of the SNP effect by the factor) is the true one, the Q_{SNP} statistic should follow the χ^2 distribution with one degree of freedom. The threshold $P < 5 \times 10^{-8}$ was chosen as the criterion for classifying a SNP as being associated with the math indicators in a manner inconsistent with acting through the quantitative factor. Only one SNP, rs13107325, had a significant Q_{SNP} statistic.

We searched for the 53 lead SNPs in the NHGRI-EBI GWAS Catalog to check whether they have been associated with other traits by previous GWAS. Using the LDtrait tool at LDlink (version 5.6.5), we searched the results of past GWAS for associations with the 53 SNPs or any variants in LD with them (defined as $r^2 > 0.6$ and within 500 kb of the focal SNP).

Genetic correlations

We also used bivariate LDSC to calculate genetic correlations between our quantitative factor and several behavioral, cognitive, and psychiatric traits: subjective well-being [23], major depressive disorder [24], autism spectrum disorder [25], cannabis use disorder [26], attention deficit hyperactivity disorder [27], bipolar disorder [28], schizophrenia [29], dyslexia [30], school grades [31], reading skills [32], neuroticism [33], externalizing [34], number of sex partners [35], number of same-sex partners [36], risk tolerance [35], smoking initiation [35], drinks per week [35], number of children [37], chronotype [38], household income [39], Townsend deprivation index [40]. Genetic correlations with height [41], brain volume [42], body mass index [43], and waist-to-hip ratio [43] were calculated as well.

In order to establish the discriminant validity of the quantitative factor, we also calculated genetic correlations with occupations assumed to be related to general and group factors in the cognitive domain. GWAS summary statistics of UKB job codes were downloaded from

http://www.nealelab.is/uk-biobank.

Statistical significance was defined as a Benjamini-Hochberg false discovery rate (FDR) less than 0.05. The P values of all genetic correlations in Supplementary Table S4 were used to calculate the FDR.

Polygenic prediction

To convert our GWAS summary statistics for the quantitative factor into weights for polygenic scores (PGS), we used the software tool PRS-CS [44]. This tool converts the univariate regression coefficients obtained in the GWAS into partial regression coefficients by plugging in a SNP covariance matrix taken from a reference panel (this step is similar to what is done by GCTA-COJO [21]) and applying Bayesian continuous shrinkage rather specifying a discrete number of prior distributions. We used the 1000 Genomes Project phase 3 participants of European descent as the reference panel.

Our validation sample consisted of the Minnesota Twin Family Study and the Sibling Interaction and Behavior Study, both of which are being conducted by the MCTFR [22]. Each complete unit in the validation sample was made up of two siblings (usually twins) and their parents. The total sample consisted of 9 067 total individuals, belonging to 2 497 family units. Details about genotyping and quality control were provided in an earlier paper [45]. Recall that because MCTFR was a part of the SSGAC meta-analysis of CP used in the main Genomic SEM, we replaced the SSGAC version of CP with the de la Fuente et al. [7] version of g ("general cognitive ability") in a secondary Genomic SEM for purposes of polygenic prediction. The genetic correlation between the quantitative factors derived in the two different ways was 0.941 (s.e. 0.046).

Our outcome measure was the third edition of the Wide Range Achievement Test (WRAT) [46]. The test has three components: Reading, Spelling, and Arithmetic. The offspring were tested on the WRAT during adolescence, between ages 13 and 21. Ten ancestry principal components, sex, and categorical age were used as covariates when regressing WRAT scores on the PGS. To deal with dependence between siblings in the same family, standard errors were clustered within family units. There were 2 641 genotyped siblings of European ancestry with available WRAT scores.

We repeated all of our PGS predictions except restricting observations to individuals with genotyped parents and adding the parental PGS as covariates. For a fixed value of the parental PGS, the PGS of the offspring vary randomly as a result of Mendelian segregation and thus provide a strong degree of causal inference [13, 47–49].

We did not apply adjustments for multiple comparisons to the predictions reported in Supplementary Table S5.

Biological annotation

We used stratified LD Score regression (S-LDSC) [17] to identify the tissues mediating the genetic effects of the SNPs affecting the quantitative factor. The S-LDSC developers recommend using so-called baseline annotations and an any-gene dummy variable as controls, and we used the 97 baseline annotations in the latest iteration (downloaded August 2023 from https://storage.googleapis.com/broad-alkesgroup-public-requester-pays/LDSCORE).

We also used the precomputed stratified LD Scores for the Genotype-Tissue Expression (GTEx) [50] data supplied by the developers [51]. S-LDSC is a general method for estimating gene-set enrichment, and here the gene set can be regarded as the top 10 percent of genes as ranked by statistical significance of the difference in expression between the focal tissue and all others. All SNPs residing inside or within 100 kb of a qualifying gene were mapped to the set. The enrichment statistic calculated by S-LDSC is

$$\frac{\text{fraction of heritability contributed by SNPs mapped to the gene set}}{\text{fraction of all SNPs mapped to the gene set}},$$

and previous experience with this method suggests that a 1.3-fold enrichment of a gene set should be regarded as a large effect size [1, 33, 51, 52]. The significance threshold $P < 0.005$ suggested by Benjamin et al. [53] was satisfied by all GTEx tissues in the category *central nervous system* and by no others (Supplementary Table S7).

To prioritize likely causal genes mediating SNP effects, we turned to Polygenic Priority Score (PoPS) [54] (downloaded October 2023 from https://github.com/FinucaneLab/pops). In summary, PoPS regresses MAGMA [55] gene-level Z statistics on various gene-level features taken from expression data, curated pathways, and the like. Note that the large number of gene-level Z statistics, roughly 20 000, ensures that the regression is likely to produce correct P values regardless of how the statistics are distributed. An initial univariate-regression step filters out features failing to meet the threshold $P <$ 0.05 with respect to predicting the gene-level statistics, and an L_2 penalty regularizes the partial regression coefficients of the surviving features. The PoP score of a gene is the dot product of its feature vector and the ridge-regression coefficients, and the gene is prioritized as likely to be causal if it has the highest PoP score of any gene in a 1-Mb locus centered on a significant lead SNP. After considering the gene features used in the PoPS paper [54], we decided to use the same expression data from mice (downloaded November 2023 from https://github.com/FinucaneLab/gene_features), the human GTEx data, and the reconstituted gene sets employed by the bioinformatic tool DEPICT [56]. We used column-mean imputation to fill in missing entries of the gene $\times$ feature matrix. In the appropriate places, we pointed to the files of gene annotations and control features supplied by the developers.

An initial PoPS run disclosed a fair number of duplicates among the DEPICT gene sets.

Only one member of each batch of duplicates was retained, according to the criteria described in an earlier paper [1]. In addition, if a batch of duplicates consisted of protein-protein interaction (PPI) subnetworks named after gene identifiers differing only in numerical suffix, we retained the lowest-numbered member. We ran PoPS again after this filtering out of duplicates. A total of 24 019 features were used in the final PoPS run for the purpose of gene prioritization.

Once in hand, the PoPS marginal and partial regression coefficients can be used as a form of gene-set enrichment analysis. We found that the top-ranked features were dominated by the gene-expression data provided by the PoPS developers, possibly because the DEPICT gene sets were missing more data. To obtain more interpretable results, we ran PoPS again except using only the DEPICT gene sets as features. We went through another round of duplicate removal and then launched another run, this one for the purpose of detecting gene-set enrichment.

Many gene sets are highly correlated (e.g., instances of *axon guidance* from different databases). To further facilitate the interpretation of the enrichment results, we applied the affinity-propagation algorithm, which segregates correlated objects into clusters and names each cluster after an exemplary member [57]. The similarity matrix was the correlation matrix of the gene sets clearing $P < 0.05$ with respect to marginal association with the MAGMA gene-level Z statistics in the DEPICT-only PoPS run. We used the `apcluster` library for `R` and retained all default settings. To give each cluster an effect size, we took the sum of its gene sets' penalized partial regression coefficients. Since the gene sets in a cluster are highly correlated, this sum approximates the contribution of the cluster to the PoP score of a gene with a feature value of unity with respect to a given member of the cluster. This procedure was closely analogous to the one described in the PoPS paper for the same purpose [54].

Determining the statistical significance of enrichment detected by this method is not an easy matter for several reasons, including the difficulty of determining the sampling distribution of a penalized regression coefficient. If a cluster has a relatively large effect size and members with very significant univariate regression coefficients (e.g., $P < 10^{-8}$), then it is not likely to be a sampling fluke. We also followed up the PoPS method with approaches yielding more conventional tests of significance, as we now describe.

To confirm the robustness of our inferences based on the DEPICT reconstituted gene sets with quantitative membership scores, we turned to two methods relying on the current discrete versions of these gene sets. The first was the PANTHER overrepresentation test, which has been implemented as a web-based tool (https://www.geneontology.org). The input to this method is a discrete list of genes supplied by the user. Our list consisted of the Ensembl identifiers of all genes prioritized by PoPS as described above. We used all default settings for analyses launched from the front page of the Gene Ontology website. Like S-LDSC, this method give an effect

size in terms of fold enrichment,

$$\frac{\text{observed \# of gene-set members in the input list}}{\text{expected \# of gene-set members in the input list}},$$

where the expectation is derived from the null hypothesis that the input gene list is a random sample of all genes in the reference gene list. All enrichments satisfying Fisher's exact test at $\mathrm{FDR} < 0.05$ were regarded as significant.

Our second method based on the discrete versions of the gene sets was the application of S-LDSC. To minimize computational burden and ensure reasonable statistical power, we restricted this analysis to non-PPI gene sets (1) clearing $\mathrm{FDR} < 0.05$ in the PoPS univariate-regression step and (2) having discrete versions with at least 200 genes according to release 2023.2 of the Molecular Signatures Database (MSigDB) (https://www.gsea-msigdb.org). We allowed a Human Phenotype gene set in MSigDB with the same human-readable identifier (e.g., *abnormal cerebral cortex morphology*) as a Mammalian Phenotype gene set to serve as a substitute. All SNPs residing inside or within 100 kb of a member gene were mapped to the set. Since these stratified LD Scores have not been precomputed by the developers, we took the gene boundaries from a GENCODE coordinate file (downloaded August 2023), taking the row in this file assuming the value of *gene* in the *feature* column as providing the stop and stop coordinates. The standard 1-centimorgan radius was used to calculate the stratified LD Scores. All enrichments satisfying $\mathrm{FDR} < 0.05$ were regarded as significant.

Code availability

We have created a repository for our analysis code and the supplementary tables at the Open Science Framework, where they can be viewed and downloaded without restriction.

References

1. Lee JJ, Wedow R, Okbay A, Kong E, Maghzian O, Zacher M, et al. Gene discovery and polygenic prediction from a genome-wide association study of educational attainment in 1.1 million individuals. Nat Genet. 2018; 50:1112–21.
2. Starr A, Riemann R. Common genetic and environmental effects on cognitive ability, conscientiousness, self-perceived abilities, and school performance. Intelligence. 2022; 93:101664.
3. Torppa M, Aro T, Eklund K, Parrila R, Eloranta AK, Ahonen T. Adolescent reading and math skills and self-concept beliefs as predictors of age 20 emotional well-being. Read Writ. 2024; 37:2075–99.
4. Neubauer AC, Pribil A, Wallner A, Hofer G. The self-other knowledge asymmetry in cognitive intelligence, emotional intelligence, and creativity. Heliyon. 2018; 4:e01061.
5. Neubauer AC, Hofer G. Self-estimates of abilities are a better reflection of individuals' personality traits than of their abilities and are also strong predictors of professional interests. Pers Indiv Differ. 2021; 169:109850.
6. Champion J, Mesa V. Factors affecting calculus completion among U. S. high school students. *The Role of Calculus in the Transition From High School to College Mathematics*. Ed. by Bressoud DM. Mathematical Association of America/National Council of Teachers of Mathematics: Washington, DC, 2017 :9–25.
7. de la Fuente J, Davies G, Grotzinger AD, Tucker-Drob EM, Deary IJ. A general dimension of genetic sharing across diverse cognitive traits inferred from molecular data. Nat Hum Behav. 2021; 5:49–58.
8. Willer CJ, Li Y, Abecasis GR. METAL: Fast and efficient meta-analysis of genomewide association scans. Bioinformatics. 2010; 26:2190–1.
9. Bulik-Sullivan B, Loh PR, Finucane HK, Ripke S, Yang J, Schizophrenia Working Group of the Psychiatric Genomics Consortium, et al. LD Score regression distinguishes confounding from polygenicity in genome-wide association studies. Nat Genet. 2015; 47:291–5.
10. Lee JJ, McGue M, Iacono WG, Chow CC. The accuracy of LD Score regression as an estimator of confounding and genetic correlations in genome-wide association studies. Genet Epidemiol. 2018; 42:783–95.
11. Rietveld CA, Medland SE, Derringer J, Yang J, Esko T, Martin NW, et al. GWAS of 126,559 individuals identifies genetic variants associated with educational attainment. Science. 2013; 340:1467–71.

12. Okbay A, Beauchamp JP, Fontana MA, Lee JJ, Pers TH, Rietveld CA, et al. Genome-wide association study identifies 74 loci associated with educational attainment. Nature. 2016; 533:539–42.

13. Okbay A, Wu Y, Wang N, Jayashankar H, Bennett M, Nehzati SM, et al. Polygenic prediction of educational attainment within and between families from genome-wide association analyses in 3 million individuals. Nat Genet. 2022; 54:437–49.

14. Grotzinger AD, Rhemtulla M, Vlaming R de, Ritchie SJ, Mallard TT, Hill WD, et al. Genomic structural equation modelling provides insights into the multivariate genetic architecture of complex traits. Nat Hum Behav. 2019; 3:513–25.

15. Bulik-Sullivan B, Finucane HK, Anttila V, Gusev A, Day FR, Loh PR, et al. An atlas of genetic correlations across human diseases and traits. Nat Genet. 2015; 47:1236–41.

16. Patterson N, Richter DJ, Gnerre S, Lander ES, Reich D. Genetic evidence for complex speciation of humans and chimpanzees. Nature. 2006; 441:1103–8.

17. Finucane HK, Bulik-Sullivan B, Gusev A, Trynka G, Reshef Y, Loh PR, et al. Partitioning heritability by functional annotation using genome-wide association summary statistics. Nat Genet. 2015; 47:1228–35.

18. Mallard TT, Linnér RK, Grotzinger AD, Sanchez-Roige S, Seidlitz J, Okbay A, et al. Multivariate GWAS of psychiatric disorders and their cardinal symptoms reveal two dimensions of cross-cutting genetic liabilities. Cell Genom. 2022; 2.

19. Chang CC, Chow CC, Tellier LC, Vattikuti S, Purcell SM, Lee JJ. Second-generation PLINK: rising to the challenge of larger and richer datasets. GigaScience. 2015; 4:7.

20. 1000 Genomes Project Consortium. A global reference for human genetic variation. Nature. 2015; 526:68–74.

21. Yang J, Ferreira T, Morris AP, Medland SE, Genetic Investigation of Anthropometric Traits Consortium, Diabetes Genetics Replication and Meta-Analysis Consortium, et al. Conditional and joint multiple-SNP analysis of GWAS summary statistics identifies additional variants influencing complex traits. Nat Genet. 2012; 44:369–75.

22. Wilson S, Haroian K, Iacono WG, Krueger RF, Lee JJ, Luciana M, et al. Minnesota Center for Twin and Family Research. Twin Res Hum Genet. 2019; 22:746–52.

23. Okbay A, Baselmans BML, De Neve JE, Turley P, Nivard MG, Fontana MA, et al. Genetic variants associated with subjective well-being, depressive symptoms, and neuroticism identified through genome-wide analyses. Nat Genet. 2016; 48:624–30.

24. Howard DM, Adams MJ, Clarke TK, Hafferty JD, Gibson J, Shirali M, et al. Genome-wide meta-analysis of depression identifies 102 independent variants and highlights the importance of the prefrontal brain regions. Nat Neurosci. 2019; 22:343–52.

25. Grove J, Ripke S, Als TD, Mattheisen M, Walters RK, Won H, et al. Identification of common genetic risk variants for autism spectrum disorder. Nat Genet. 2019; 51:431–44.

26. Johnson EC, Demontis D, Thorgeirsson TE, Walters RK, Polimanti R, Hatoum AS, et al. A large-scale genome-wide association study meta-analysis of cannabis use disorder. Lancet Psychiatry. 2020; 7:1032–45.

27. Demontis D, Walters GB, Athanasiadis G, Walters R, Therrien K, Nielsen TT, et al. Genome-wide analyses of ADHD identify 27 risk loci, refine the genetic architecture and implicate several cognitive domains. Nat Genet. 2023; 55:198–208.

28. Mullins N, Forstner AJ, O'Connell KS, Coombes B, Coleman JRI, Qiao Z, et al. Genome-wide association study of more than 40,000 bipolar disorder cases provides new insights into the underlying biology. Nat Genet. 2021; 53:817–29.

29. Pardiñas AS, Holmans PA, Pocklington AJ, Escott-Price V, Ripke S, Carrera N, et al. Common schizophrenia alleles are enriched in mutation-intolerant genes and in regions under strong background selection. Nat Genet. 2018; 50:381–9.

30. Doust C, Fontanillas P, Eising E, Gordon SD, Wang Z, Alagöz G, et al. Discovery of 42 genome-wide significant loci associated with dyslexia. Nat Genet. 2022; 54:1621–9.

31. Rajagopal VM, Ganna A, Coleman JR, Allegrini A, Voloudakis G, Grove J, et al. Genome-wide association study of school grades identifies genetic overlap between language ability, psychopathology and creativity. Sci Rep. 2023; 13:429.

32. Eising E, Mirza-Schreiber N, De Zeeuw EL, Wang CA, Truong DT, Allegrini AG, et al. Genome-wide analyses of individual differences in quantitatively assessed reading- and language-related skills in up to 34,000 people. Proc Natl Acad Sci USA. 2022; 119:e2202764119.

33. Kim Y, Saunders GRB, Giannelis A, Willoughby EA, DeYoung CG, Lee JJ. Genetic and neural bases of the neuroticism general factor. Biol Psychol. 2023; 184:108692.

34. Williams CM, Poore H, Tanksley PT, Kweon H, Courchesne-Krak NS, Londono-Correa D, et al. Guidelines for evaluating the comparability of down-sampled GWAS summary statistics. Behav Genet. 2023; 53:404–15.

35. Linnér RK, Biroli P, Kong E, Meddens SFW, Wedow R, Fontana MA, et al. Genome-wide association analyses of risk tolerance and risky behaviors in over 1 million individuals identify hundreds of loci and shared genetic influences. Nat Genet. 2019; 51:245–57.

36. Ganna A, Verweij KJH, Nivard MG, Maier R, Wedow R, Busch AS, et al. Large-scale GWAS reveals insights into the genetic architecture of same-sex sexual behavior. Science. 2019; 365:eaat7693.

37. Barban N, Jansen R, de Vlaming R, Vaez A, Mandemakers J, Tropf FC, et al. Genome-wide analysis identifies 12 loci influencing human reproductive behavior. Nat Genet. 2016; 48:1462–72.

38. Jones SE, Lane JM, Wood AR, Hees VT van, Tyrrell J, Beaumont RN, et al. Genome-wide association analyses of chronotype in 697,828 individuals provides insights into circadian rhythms. Nat Commun. 2019; 10:343.

39. Hill WD, Davies NM, Ritchie SJ, Skene NG, Bryois J, Bell S, et al. Genome-wide analysis identifies molecular systems and 149 genetic loci associated with income. Nat Commun. 2019; 10:5741.

40. Hill WD, Hagenaars SP, Marioni RE, Harris SE, Liewald DC, Davies G, et al. Molecular genetic contributions to social deprivation and household income in UK Biobank. Curr Biol. 2016; 26:3083–9.

41. Yengo L, Vedantam S, Marouli E, Sidorenko J, Bartell E, Sakaue S, et al. A saturated map of common genetic variants associated with human height. Nature. 2022; 610:704–12.

42. Jansen PR, Nagel M, Watanabe K, Wei Y, Savage JE, de Leeuw CA, et al. Genome-wide meta-analysis of brain volume identifies genomic loci and genes shared with intelligence. Nat Commun. 2020; 11:5606.

43. Pulit SL, Stoneman C, Morris AP, Wood AR, Glastonbury CA, Tyrrell J, et al. Meta-analysis of genome-wide association studies for body fat distribution in 694 649 individuals of European ancestry. Hum Mol Genet. 2019; 28:166–74.

44. Ge T, Chen CY, Ni Y, Feng YCA, Smoller JW. Polygenic prediction via Bayesian regression and continuous shrinkage priors. Nat Commun. 2019; 10:1776.

45. Miller MB, Basu S, Cunningham J, Eskin E, Malone SM, Oetting WS, et al. The Minnesota Center for Twin and Family Research genome-wide association study. Twin Res Hum Genet. 2012; 15:767–74.

46. Snelbaker AJ, Wilkinson GS, Robertson GJ, Glutting JJ. Wide Range Achievement Test 3 (WRAT 3). *Understanding Psychological Assessment*. Ed. by Dorfman WI, Hersen M. Springer: New York, 2001 :259–74.

47. Fisher RA. Statistical methods in genetics. Heredity. 1952; 6:1–12.

48. Laird NM, Lange C. Family-based designs in the age of large-scale gene-association studies. Nat Rev Genet. 2006; 7:385–94.

49. Lee JJ, Chow CC. The causal meaning of Fisher's average effect. Genet Res. 2013; 95:89–109.

50. GTEx Consortium. The Genotype-Tissue Expression (GTEx) pilot analysis: Multitissue gene regulation in humans. Science. 2015; 348:648–60.

51. Finucane HK, Reshef YA, Anttila V, Slowikowski K, Gusev A, Byrnes A, et al. Heritability enrichment of specifically expressed genes identifies disease-relevant tissues and cell types. Nat Genet. 2018; 50:621–9.

52. Kim SS, Dai C, Hormozdiari F, Geijn B van de, Gazal S, Park Y, et al. Genes with high network connectivity are enriched for disease heritability. Am J Hum Genet. 2019; 104:896–913.

53. Benjamin DJ, Berger JO, Johannesson M, Nosek BA, Wagenmakers EJ, Berk R, et al. Redefine statistical significance. Nat Hum Behav. 2018; 2:6–10.

54. Weeks EM, Ulirsch JC, Cheng NY, Trippe BL, Fine RS, Miao J, et al. Leveraging polygenic enrichments of gene features to predict genes underlying complex traits and diseases. Nat Genet. 2023; 55:1267–76.

55. de Leeuw CA, Mooij JM, Heskes T, Posthuma D. MAGMA: Generalized gene-set analysis of GWAS data. PLoS Comput Biol. 2015; 11:e1004219.

56. Pers TH, Karjalainen J, Chan Y, Westra HJ, Wood AR, Yang J, et al. Biological interpretation of genome-wide association studies using predicted gene functions. Nat Commun. 2015; 6:5890.

57. Frey BJ, Dueck D. Clustering by passing messages between data points. Science. 2007; 315:972–6.
